# Many Models, Little Adoption—What Accounts for Low Uptake of Machine Learning Models for Atrial Fibrillation Prediction and Detection?

**DOI:** 10.3390/jcm13051313

**Published:** 2024-02-26

**Authors:** Yuki Kawamura, Alireza Vafaei Sadr, Vida Abedi, Ramin Zand

**Affiliations:** 1 School of Clinical Medicine, University of Cambridge, Cambridge CB3 0SP, UK; 2 Department of Public Health Sciences, College of Medicine, The Pennsylvania State University, Hershey, PA 17033, USAvabedi@pennstatehealth.psu.edu (V.A.); 3 Department of Neurology, College of Medicine, The Pennsylvania State University, Hershey, PA 17033, USA

**Keywords:** machine learning, atrial fibrillation, prevention, detection, stroke, neural networks, decision trees, artificial intelligence

## Abstract

(1) **Background:** Atrial fibrillation (AF) is a major risk factor for stroke and is often underdiagnosed, despite being present in 13–26% of ischemic stroke patients. Recently, a significant number of machine learning (ML)-based models have been proposed for AF prediction and detection for primary and secondary stroke prevention. However, clinical translation of these technological innovations to close the AF care gap has been scant. Herein, we sought to systematically examine studies, employing ML models to predict incident AF in a population without prior AF or to detect paroxysmal AF in stroke cohorts to identify key reasons for the lack of translation into the clinical workflow. We conclude with a set of recommendations to improve the clinical translatability of ML-based models for AF. (2) **Methods**: MEDLINE, Embase, Web of Science, Clinicaltrials.gov, and ICTRP databases were searched for relevant articles from the inception of the databases up to September 2022 to identify peer-reviewed articles in English that used ML methods to predict incident AF or detect AF after stroke and reported adequate performance metrics. The search yielded 2815 articles, of which 16 studies using ML models to predict incident AF and three studies focusing on ML models to detect AF post-stroke were included. (3) **Conclusions**: This study highlights that (1) many models utilized only a limited subset of variables available from patients’ health records; (2) only 37% of models were externally validated, and stratified analysis was often lacking; (3) 0% of models and 53% of datasets were explicitly made available, limiting reproducibility and transparency; and (4) data pre-processing did not include bias mitigation and sufficient details, leading to potential selection bias. Low generalizability, high false alarm rate, and lack of interpretability were identified as additional factors to be addressed before ML models can be widely deployed in the clinical care setting. Given these limitations, our recommendations to improve the uptake of ML models for better AF outcomes include improving generalizability, reducing potential systemic biases, and investing in external validation studies whilst developing a transparent modeling pipeline to ensure reproducibility.

## 1. Introduction

### 1.1. Background

A prominent risk factor for stroke is atrial fibrillation (AF), which increases the incidence of stroke by between 2.6- and 4.5-fold depending on decade of life [1] and recurrence of stroke by around 2-fold [2]. Given that 13–26% of patients with ischemic stroke have AF [3], prediction and management of AF hold promise in addressing the disease burden of stroke. Yet, screening and diagnosis of AF are not straightforward, especially amongst asymptomatic patients. In fact, 13.1% of patients with AF in the United States are estimated to be undiagnosed, with 56% of undiagnosed patients belonging to higher stroke risk groups with a CHADS_2_ score of 2 and above [4]. 

AF monitoring is complicated by the fact that up to a third of patients do not experience symptoms [5], which reduces the pretest probability. In absence of any symptomatic cues, AF episodes can only be captured through continuous long-term monitoring given their unpredictable nature, which can generate copious data. In addition, although Holter monitors are frequently used for long-term monitoring of AF, patient compliance is often a limiting factor for longer monitoring durations [6], possibly due to their bulkiness and need for applied leads. These challenges can hinder diagnosis of AF and initiation of anticoagulants for thromboembolism prevention, which is problematic given that recent studies have shown that AF can be detected after longer monitoring in between 20 and 30% of patients diagnosed with cryptogenic strokes [7,8,9]. 

Big data and machine learning (ML) algorithms can improve AF prediction and diagnosis in terms of both accuracy and throughput. Digital health approaches could especially be effective in AF monitoring, since relevant measurements including heart rate and ECGs can be obtained using wearable devices which are readily available, noninvasive, and need not be replaced regularly in contrast to, for example, continuous glucose monitors. Indeed, the low uptake barrier for utilizing wearables for AF monitoring is illustrated by the fact that clinical trials investigating the performance of wearables such as the Apple Watch^®^ [10] and Fitbit^®^ [11] were able to recruit upwards of 400,000 volunteers. Vast amounts of data generated by such wearables, coupled with the ability of ML algorithms to model complex datasets, can enhance thromboembolic stroke prevention primarily in two ways. Screening a general population and predicting incident AF can allow high-risk patients to be monitored more carefully for the primary occurrence of embolic stroke. In addition, continuous electrocardiogram (ECG) monitoring of patients after ischemic stroke can identify patients for whom anticoagulants should be started to prevent stroke recurrence. Hence, improved prediction and detection capabilities conferred via ML models can improve primary and secondary stroke prevention.

Despite potential advantages, however, clinical uptake of ML-based models has been slow, and only six devices for artificial intelligence (AI)-based AF detection have been approved in the United States and Europe between 2015 and 2020 [12]. This study therefore aims to characterize the barriers to incorporating ML models into the clinical workflow of AF management. We performed a scoping review of studies proposing or evaluating ML models for AF prediction and detection to illustrate the current state of the art and analyzed the limitations of each of the studies, which might hinder clinical uptake. Given the observed limitations, we propose several recommendations for future studies that could promote the clinical translation of ML models for AF care. 

### 1.2. Scope and Key Questions

We sought to address the following key questions (KQ):

(KQ1) In adult patients without a known history of stroke or AF or cardiovascular comorbidities, what are the performance statistics, data features and processing steps, and limitations of ML models in predicting incidence of AF?
(KQ2) In adult patients with a previous history of stroke, what are the performance statistics, data features and processing steps, and limitations of ML models for AF detection?
A PICOTS (population, interventions, comparators, outcomes, timing, and setting) table with details on the key questions is shown below (Table 1).

## 2. Methods

### 2.1. Search Strategy

The scoping review was performed and written in accordance with the Enhancing the QUAlity and Transparency of Health Research (EQUATOR) and Preferred Reporting Items for Systematic Reviews and Meta-Analyses (PRISMA; Table S1) guidelines. MEDLINE, Embase, Web of Science, Clinicaltrials.gov, and ICTRP databases were searched for articles proposing or evaluating ML models (including tree-based models but not logistic regression models) to (1) predict the incidence of atrial fibrillation in a population with no known history of stroke or atrial fibrillation or (2) to detect atrial fibrillation in a population with a previous history of stroke. Original research articles in English published in peer-reviewed journals were included. Conference abstracts were also included in the search but were removed as duplicates if the same model was evaluated by the same group in a subsequent peer-reviewed article (i.e., if they were preliminary results for a subsequent published article). Databases were searched for manuscripts containing variants of “atrial fibrillation”, “stroke”, and “machine learning/artificial intelligence”, combined with “predict” or “detect” as appropriate. Details of search terms can be found in the Supplementary Information. 

### 2.2. Eligibility Criteria

Observational cohort studies evaluating the accuracy of ML models predicting the incidence of atrial fibrillation in an adult population without a history of AF or detecting AF in a stroke population in any setting were included. We selected studies that provided an area under the receiver operating characteristics curve (AUC) or data sufficient to create a 2 × 2 table and used the diagnosis of AF using ECG or discharge codes as a gold standard. For studies regarding the detection of AF in a stroke population, only those that defined previous incidence of stroke based on the diagnosis of stroke by a neurologist or hospitalist or discharge codes were included. 

Studies using or proposing new clinical risk scores, studies using mortality rather than diagnosis as the endpoint, studies performed on a disease population with underlying cardiovascular comorbidities (such as chronic kidney disease or diabetes) or structural heart disease, and studies not authored in English were excluded. 

### 2.3. Data Collection

Data extraction was performed in duplicate. After the removal of irrelevant records, screening was performed by two authors (YK, RZ) to identify studies that met the inclusion and exclusion criteria. Disagreements were resolved through discussion involving a third reviewer (VA). Full-text reviews were performed for selected studies to identify model architecture, input data and list of variables, study population, data pre-processing including addressing missingness and potential selection bias, validation, results including model performance metrics, follow-up period, and availability of models and codes for reproducing and transparency. Model performance was rounded to two significant figures. 

## 3. Results

### 3.1. AI for Primary Stroke Prevention: Prediction of Atrial Fibrillation in the General Population

#### 3.1.1. Search Results and Study Characteristics

**Figure 1:**
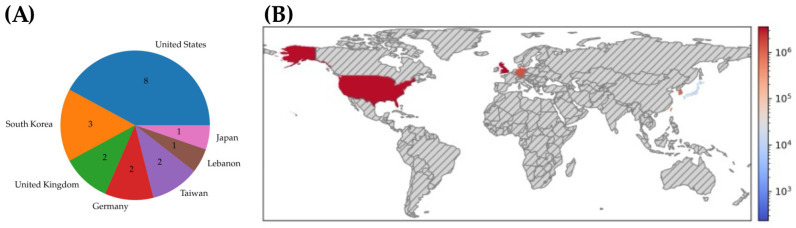
Cohort Characteristics of Selected Studies. (**A**) Number of selected studies performed in each country, (**B**) World map color-coded by study population size.

The search yielded 2234 results. After removing duplicates, 1352 studies were screened based on their title and abstracts, which resulted in the selection of 129 studies for further screening. Of these studies, full texts for three studies were not retrieved. After reviewing the full texts of 126 studies, 16 studies [13,14,15,16,17,18,19,20,21,22,23,24,25,26,27,28] met the inclusion/exclusion criteria (Figure S1, Table S2). The 16 identified studies were conducted in seven different countries: Seven in the United States, three in Korea, two in the United Kingdom, and one in Japan, Lebanon, Germany, and Taiwan (Figure 1, Table S3). The publication dates spanned five years, ranging from 2017 to 2022. Most of the studies were conducted retrospectively (14/16), whereas two were conducted prospectively in the United States. The majority of studies (10/16) curated their data in a database accessible to approved investigators.

#### 3.1.2. Machine Learning Models: Characteristics and Performance Metrics

**Figure 2:**
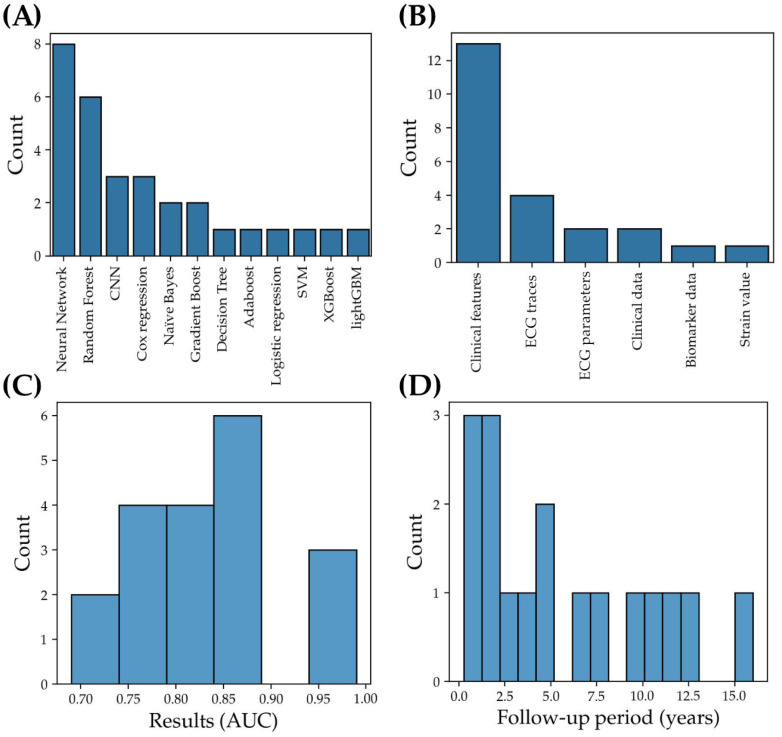
Model Characteristics of Selected Studies. (**A**) number of studies (count) for different model architectures, (**B**) number of studies (count) for different input data types, (**C**) number of studies (count) versus model AUC, and (**D**) number of studies (count) for different follow-up periods of selected studies.

Of the 16 studies, two studies were validation studies on the same neural network-based model incorporating clinical variables, whereas two studies were validation studies of a separate convolutional neural network model (ECG-AI) developed for AF detection but repurposed for prediction (Table S4). Hence, the search yielded 14 different prediction models. Half (7/14) of the models used tree-based models, whereas 11 of the models used neural network-based models (Figure 2A). Of the tree-based models, five were random forest models, with the rest being Adaboost and gradient-boosted trees (including lightGBM and XGBoost). Only four of the models directly took ECG traces as an input for a convolutional neural network (CNN) model, but two additional studies incorporated ECG parameters (such as R-R intervals) in the analysis (Figure 2B, Table S4). In some cases, probability outputs from a CNN model predicting AF were used as input into a Cox regression model together with clinical data. Other model architectures used included support vector machines. The performance of predictive models as reported by the authors ranged from an AUC of 0.69 to 0.96 for simple neural network models, 0.72 to 0.84 for CNN-based neural networks, and 0.75 to 0.99 for tree-based models (Figure 2C, Table S4). The follow-up period ranged from 3 months to 16 years (Figure 2D, Table S3).

#### 3.1.3. Analysis of Limiting Factors and Best Practices: From Data Pre-Processing to Model Validation

**Figure 3:**
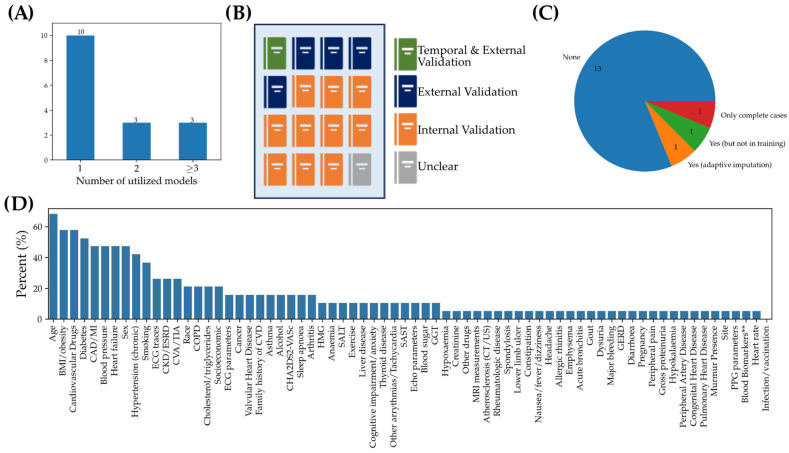
Data Processing in Selected Studies. (**A**) number of studies evaluating 1, 2, or more than 3 models, (**B**) type of validation performed in each of the 16 studies analyzed, (**C**) number of studies reporting sparse data processing, and (**D**) percent of studies for each input feature.

Multiple factors limiting the real-world implementation of AI models for prediction and detection were identified (Figure 3, Table 2). One of the limiting factors was the lack of performance benchmarking against conventional predictive models, with the majority of studies (10/16) utilizing only one model architecture without comparing the performance of ML models against baseline models such as logistic regression (Figure 3A). The lack of external validation was another common shortfall, with only a subset (5/16) of studies reporting external validation and most of the studies relying on internal validation (Figure 3B). Furthermore, reporting of pre-processing to mitigate sparseness was limited, with only one study reporting adaptive imputation, two studies reporting complete case selection, and the other studies with no mention of pre-processing for sparseness (Figure 3C). In addition, the identified models often did not fully make use of the myriad data features that were available and instead used a limited subset of these features (Figure 3D, Table S5). Finally, none of the studies selected made their model or code available in public repositories (Table S4). 

### 3.2. AI for Secondary Stroke Prevention: Detection of Atrial Fibrillation in Stroke Cohorts 

#### 3.2.1. Search Results and Study Characteristics

The search yielded 581 articles. After removal of duplicates and further screening, three studies [30,31,32] were included in the review. Details on the study selection are summarized in Figure S2 and Table S6. Of the three identified studies, one was performed in Germany in 2014, one in the United States in 2019, and one in Taiwan in 2014 (Table S7). All the studies were performed prospectively on patients with ischemic stroke but differed slightly in their criteria, which are summarized in Table S7. None of the studies uploaded their data in a public repository. 

#### 3.2.2. Machine Learning Models: Characteristics and Performance Metrics 

Of the three studies, two were validation studies on models developed previously, whereas one study proposed a new model (Table 3). The models were trained on R-R intervals in two of the studies, whereas one study used ECG traces directly (Table 3). The model architectures used were support vector machines (*n* = 2) and convolutional neural networks (*n* = 1). The sensitivity of detection models was between 63 and 95%, whereas the specificity was between 35 and 96%. Reported positive predictive values ranged between 23 and 27%, whereas negative predictive values ranged between 94 and 96%. 

#### 3.2.3. Analysis of Limiting Factors and Best Practices: From Data Pre-Processing to Model Validation

Limitations identified by the study authors included the limited size and setting of the cohort and limited monitoring duration (Table 4). Other limitations included low positive predictive value and low sensitivity. Furthermore, whilst two of the studies validated previously published models, one of the studies used a proprietary model whose development was not available. Common shortfalls regarding model training included the non-generalizability of the training cohort, as well the as lack of external validation. Other shortfalls concerned the quality of trained models, such as only marginal improvement on existing clinical risk scores and low positive predictive values (PPV). Additional limiting factors that could hinder real-life implementation included the lack of interpretability. 

## 4. Discussion 

### 4.1. Recommendations for Clinical Implementation of ML Models 

Our study identified 19 studies proposing or validating ML-based models for the prediction and detection of AF. This review contributes insights into clinically relevant topics with limited prior attention, such as the reasons for low clinical translation of ML models as well as ML models for post-stroke AF detection. Whilst the results underscored the ability of ML models to outperform clinical risk scores and logistic regression, analyzing the studies revealed limitations in study design and model construction that negatively affected generalizability and thus could affect clinical translation. In particular, we identified four concerns which could most significantly hinder clinical uptake and provide recommendations for each below. 

Firstly, many of the ML models did not include a full picture of the patients’ health status and demographic information potentially available in electronic health records and instead used a limited subset of data features. Furthermore, information regarding imaging, laboratory values, and blood biomarkers were not frequently included as input variables in the model. Including multimodal information as inputs in ML models could better aid clinicians in decision-making as it more closely mimics diagnostic reasoning and can also improve model performance. We suggest that future models take advantage of multimodal data available in generating a more holistic and clinically relevant decision. 

Second, robust external validation should be performed to improve model generalizability. Given that complex ML models often overfit training data, robust external validation is essential in ensuring reliable results. However, only a small subset of selected studies included external validation, with the majority of studies relying on internal validation using data with the same demographic characteristics as the training set. Clinicians might be disinclined to adopt algorithms lacking external validation, since they might consider the evidence base to be less rigorous in comparison to clinical evidence based on consensus derived from multiple large trials. Decentralized training methods, such as federated learning and swarm learning, hold potential promise in surmounting privacy concerns whilst ensuring models are appropriately trained and tested on cohorts across multiple health systems [34,35]. We recommend that future studies incorporate external validation or use decentralized training methods to ensure that the ML model generates reliable results on unseen cohorts. 

Thirdly, improving code, model, and data availability is essential to ensure transparency and promote clinicians’ confidence in using ML-based models. Indeed, our analysis highlighted the disparity in cohort characteristics such as AF prevalence and ethnic composition as well as input variables and data formats amongst different studies, all of which can hinder generalizability. The availability of both the source code and data in a readily accessible and standardized fashion is necessary to promote models that can be deployed widely and effectively. Yet, none of the reviewed studies made their source code or even a black-box model publicly available online. Additionally, whilst the majority of studies on AF prediction curated their data in a dataset accessible to approved investigators, none of the studies for AF detection curated their data in such databases. Importantly, only one of the studies curated its data in a standardized dataset format, such as the Observational Medical Outcomes Partnership (OMOP) Common Dataset model [36], or the Patient-Centered Outcomes Research (PCORnet) Common Data Model (CDM) [37]. We recommend that publishing requirements strongly encourage the source code or the trained model to be made available, as well as de-identified data where appropriate. 

Finally, care must be taken in data pre-processing and the deployment of ML models so that existing biases in healthcare are not unwittingly perpetuated. Indeed, a commercially available model in current use falsely assigned lower risk values to Black patients because it was trained using healthcare costs as a proxy for the severity of condition, despite the fact that on average less healthcare costs are spent on Black patients [38]. Clinical use of certain ML models might also require patients to have access to digital devices, which can be a hurdle for some high-risk populations including older patients and underprivileged patients [39], who risk being excluded from valuable studies. Possible biases were also identified in our analysis, such as the fact that most of the studies were performed in developed countries with high volumes of digital data, as well as selection bias for patients able to afford tertiary care, especially in the United States. Asian and Caucasian populations were well-represented, but no studies were performed in Africa or South America. We suggest maximizing diversity in training cohorts, incorporating fairness audits as standard practice, and post-processing to mitigate bias and ensure that ML models are amenable to use in a wide variety of contexts. 

### 4.2. Further Considerations for ML Models in Clinical Practice 

Effective incorporation of ML models in clinical practice requires not only the availability of high-performing and generalizable models but also support for both physicians and patients to reap maximal benefits. For example, the nature of tort law which privileges standard of care regardless of its effectiveness in a particular case could put physicians at risk of liability should care be withheld based on risk assessment using ML models [40]. Implementing a clear legal framework on the use of and liability regarding ML models in clinical practice will aid physicians in defining how to incorporate model output in the clinical workflow and is likely to boost their uptake. Support for patients is just as important; a study comparing five commercially available wearable devices for AF monitoring demonstrated that approximately 20% of photoplethysmography or ECG traces were inconclusive due to artefact, which was a much higher rate than the rate published by the manufacturers [41]. Educating patients and promoting awareness of correct device use could be effective in improving the quality of collected data. Clinical trials validating the real-world effectiveness of ML-guided interventions would provide insights into ways in which human-AI interactions can be optimized. 

Additionally, cost-effectiveness for the implementation of AF screening must also be considered. Given that AF prediction for a general population requires no invasive investigation and stroke patients with suspicion of cardioembolic stroke already receive telemetry, implementation of ML models itself might not be a significant economic burden. An analysis using the prediction model proposed by Hill et al., 2019 [16] suggested that AF screening will increase costs by approximately GBP 322 million but will also result an increase of 81,000 QALYs [42] in the UK population. However, screening a wide population, especially with a low pre-test probability of AF, could lead to an increased burden on the healthcare system due to more visits and confirmatory testing in a predominantly healthy, younger population. Indeed, the 2023 ACC/AHA/ACCP/HRS guidelines state that it has not been established that patients deemed to be of high risk of developing AF by validated risk scores benefit from screening and interventions [43]. Choosing the right target population and creating a clinical and legal framework which avoids the need for additional follow-up clinician visits are ways in which cost-effectiveness could be improved. 

### 4.3. Limitations of the Study

This study has several limitations. First, only a limited number of studies were found regarding the detection of AF in stroke patients, because whilst there were many algorithms tested on a general population, there were few which were specifically tested on a stroke cohort. Future studies could externally validate existing algorithms on stroke cohorts to evaluate the performance of ML models on this clinically relevant population. Second, a quantitative comparison of different models was precluded because of significant differences in the types of models and variations in the input data, making it impossible to compare the performances of the models directly with one another. Studies comparing the performance of multiple models on an identical unseen dataset could provide insights into the generalizability of currently available ML models. 

### 4.4. Conclusions

In conclusion, ML models hold promise in accurately predicting AF in a healthy population for primary prevention or detecting AF in a stroke population for secondary stroke prevention. Yet, their real-world deployment is currently limited. Our recommendations to improve clinical translation include improving generalizability, reducing potential systemic biases, and investing in external validation studies whilst developing a transparent modeling pipeline to ensure reproducibility. Future developments addressing these points would facilitate the much-needed translation of these models into the clinic. 

## Figures and Tables

**Table 1:** PICOTS (population, interventions, comparators, outcomes, timing, and setting) table for Key Questions addressed in the scoping review.

	Key Question 1	Key Question 2
Population	Adult patients without a known history of stroke or atrial fibrillation or cardiovascular comorbidities	Adult patients with a previous history of stroke
Interventions	ML models to predict incidence of atrial fibrillation	ML models to detect atrial fibrillation
Comparators	None	None
Outcomes	Predictive performance of models (AUC or 2 × 2 table) Input data features and data processing steps used in model development Limitations hindering model incorporation into clinical practice, including those listed by authors	Detection performance of models (AUC or 2 × 2 table) Input data features and data processing steps used in model development Limitations hindering model incorporation into clinical practice, including those listed by authors
Timing	Any observational cohort study	Any observational cohort study
Setting	Any setting	Any setting

**Table 2:** Limitations of selected models for AF prediction.

Study (Original Study Proposing Model If Validation Study)	Limitations of the Study Suggested by the Authors	Additional Limitations
Ahmad et al., 2020 [13]	None listed	Extremely small sample size No external validation
Ambale-Venkatesh et al., 2017 [14]	Patient cohort might not be representative of general population Did not include genetic data Longitudinal changes in risk not considered	No external validation
ECG-AI (Attia et al., 2019 [29], Christopoulos et al., 2020 [15], Kaminski et al., 2022 [20])	Lack of interpretability makes it difficult to direct therapy Patient cohort (training and external validation) might not be representative of general population Datasets could have been mislabeled	Low PPV (3.2% at 95% sensitivity threshold) *
Hill et al., 2019 [16], Sekelj et al., 2021 [27]	Improvement in accuracy was small Patient cohort might not be representative of general population No accounting for ethnic differences Datasets could have been mislabeled Potentially low cost-effectiveness	Low PPV (12% at 75% sensitivity threshold)
Hirota et al., 2021 [17]	Patient cohort might not be generalizable ECG signals might not be generalizable to other devices	No external validation
Hu et al., 2019 [18]	Possibility of unaccounted confounding factors Lacking information about lifestyle or family history of AF	No external validation
Joo et al., 2020 [19]	None listed	No external validation Relatively low AUC
Khurshid et al., 2022 [21]	Potential selection bias Limited sample size Prediction window was too long/too short Potential lack of interpretability	Calibration of analysis demonstrates relatively mediocre performance of AI model in isolation (when not combined with CHARGE-AF)
Kim et al., 2020 [22]	Potential selection bias Limited precision regarding time of AF occurrence Possible confounding variables No external validation	
Kim et al., 2020 [23]	Model only marginally outperformed clinical risk score when using same number of inputs	No external validation
Lip et al., 2022 [24]	Potential selection bias	No external validation
Raghunath et al., 2021 [25]	Limited AF monitoring could have led to missed AF occurrences. Limited time of AF recording Patient cohort might not be representative of general population Lack of interpretability makes it difficult to find pathophysiological basis of prediction and establish causality	No true external validation
Schnabel et al., 2023 [26]	Patient cohort might not be representative of general population	Low PPV (13% for 95% sensitivity threshold)
Tiwari et al., 2020 [28]	Low sensitivity Machine learning model did not significantly outperform logistic regression model and does not work in real time No time-varying effects measured Imprecise recording of when AF occurred Did not incorporate biomarkers or laboratory values Low PPV (5.9% at 75% sensitivity threshold) Patient cohort might not be representative of general population	

PPV: Positive predictive value. * Low PPV is listed as a limitation only when their values are provided; this does not suggest that studies for which this limitation is not stated have higher PPVs.

**Table 3:** Characteristics of Models for Detection of Atrial Fibrillation in a Stroke Population.

Selected Study (Original Study Proposing Model If Validation Study)	Input Data	Data Source/Data Curated for Approved Access?	Model Architecture/Validation	Results	Model Interpretation	Code or Model Available/Reported Handling of Sparse Data	Model Currently Available for Clinical Use?
Rabinstein et al., 2021 [32] (ECG-AI [29])	ECG trace	Prospective; local EHR/no	CNN/External	Sn: 63% Sp: 75% PPV: 23% NPV: 94%	No	Neither/No	No
Reinke et al., 2018 [30]/ (Schaefer et al., 2014 [33])	ECG parameters	Prospective; local EHR/no	SVM (Proprietary model)/External	Sn: 95% Sp: 35% PPV: 27% NPV: 96%	No	Neither/No	Yes
Shan et al., 2014 [31]	Photoplethysmogram data	Prospective; local EHR/no	SVM/Internal	Acc: 96% Sn: 94% Sp: 96% AUC: 0.97	No	Neither/No	No

Sn: Sensitivity; Sp: Specificity; PPV: Positive predictive value; NPV: Negative predictive value; AUC: Area under the curve.

**Table 4:** Limitations of Selected Models for AF Detection.

Study	Limitations of the Study Suggested by the Authors	Additional Limitations
Rabinstein et al., 2021 [32]	Small patient cohort Short duration for AF monitoring Reduced performance when adjusted for age	Low sensitivity (63%) Low PPV (23%) *
Reinke et al., 2018 [30]	Monocentric design and small cohort Proprietary algorithm which has not been thoroughly field-tested Limited use of multimodal data	Low PPV (27%) No parameters/intermediate weights available (black-box model)
Shan et al., 2014 [31]	None listed	No external validation

PPV: Positive predictive value. * Low PPV is listed as a limitation only when their values are provided; this does not suggest that studies for which this limitation is not stated have higher PPVs.

## Data Availability

No new data were created or analyzed in this study. Data sharing is not applicable to this article.

## Supplementary Materials

The following supporting information can be downloaded at: https://www.mdpi.com/article/10.3390/jcm13051313/s1, Table S1. PRISMA checklist. Table S2. Excluded Studies for Prediction of Incident Atrial Fibrillation in Population without Prior AF. Table S3. Population Characteristics of Studies for Prediction of Atrial Fibrillation in General Population. Table S4. Characteristics of Models for Prediction of Atrial Fibrillation in the General Population. Table S5. Features Used in Final Model Training. Table S6. Excluded Studies for Detection of Atrial Fibrillation after Stroke. Table S7. Population Characteristics of Studies for Detection of Atrial Fibrillation in a Stroke Population. Figure S1. PRISMA Flow Diagram of Studies Describing Prediction of Incident Atrial Fibrillation in a Population without Prior AF. Figure S2. PRISMA Flow Diagram of Studies Describing Detection of Atrial Fibrillation in a Stroke Population.
