# Supplementary material for: Many Models, Little Adoption—What Accounts for Low Uptake of Machine Learning Models for Atrial Fibrillation Prediction and Detection?
Source: J Clin Med. 2024 Feb 26;13(5):1313. doi: 10.3390/jcm13051313 (PMC10932407; doi:10.3390/jcm13051313)
Supplement: Supplementary file 1 [file jcm-13-01313-s001.zip › jcm-2852354-supplementary.pdf]

Many Models, Little Adoption – What Accounts for Low Uptake of Machine Learning Models for Atrial Fibrillation Prediction and Detection?

Supplementary Information

Yuki Kawamura [1,*], Alireza Vafaei Sadr [2], Vida Abedi [2] and Ramin Zand [3,*]

[1] School of Clinical Medicine, University of Cambridge, Cambridge CB3 0SP, UK
[2] Department of Public Health Sciences, College of Medicine, The Pennsylvania State University,
Hershey, PA 17033, USA; vabedi@pennstatehealth.psu.edu (V.A.)
[3] Department of Neurology, College of Medicine, The Pennsylvania State University, Hershey, PA 17033, USA
* Correspondence: yk402@cam.ac.uk (Y.K.); rzand@pennstatehealth.psu.edu (R.Z.)

Keywords
machine learning; atrial fibrillation; prevention; detection; stroke; neural networks; decision trees; paroxysmal

Search Terms – Prediction of AF in Healthy Population

Ovid MEDLINE(R) and Epub Ahead of Print, In-Process, In-Data-Review and Other Non-Indexed Citations, Daily and Versions <1946 to September 20, 2022>

1 (af or atrial fibrillation or atrial flutter*).ti,ab,kw. 105677
2 atrial fibrillation/ or atrial flutter/ 70394
3 predict*.ti,ab,kw. 1917375
4 (machine learning or deep learning or neural network* or ai or artificial intelligence or decision tree or gradient boost* or XGBoost or Catboost or Bayes or SVM or support vector machine or random forest).ti,ab,kw. 228579
5 artificial intelligence/ or machine learning/ or deep learning/ or supervised machine learning/ 71951
6 1 or 2 118611
7 4 or 5 247461
8 3 and 6 and 7
437 results

Embase <1974 to 2022 September 20>

1 (af or atrial fibrillation or atrial flutter*).ti,ab,kw. 185112
2 atrial fibrillation/ or atrial flutter/ 102049
3 predict*.ti,ab,kw. 2566260
4 (machine learning or deep learning or neural network* or ai or artificial intelligence or decision tree or gradient boost* or XGBoost or Catboost or Bayes or SVM or support vector machine or random forest).ti,ab,kw. 279848
5 artificial intelligence/ or machine learning/ or deep learning/ or supervised machine learning/ 130437
6 1 or 2 212881
7 4 or 5 307847
8 3 and 6 and 7
886 results

Web of Science
af or atrial fibrillation or atrial flutter* (Topic) and predict* (Topic) and machine learning or deep learning or neural network* or ai or artificial intelligence or decision tree or gradient boost* or XGBoost or Catboost or Bayes or SVM or support vector machine or random forest (Topic) 798 results

WHO ICTRP
("Atrial Fibrillation" OR "Atrial flutter") AND (predict or prediction) 44 results

Clinicaltrials.gov
("Atrial Fibrillation" OR "Atrial flutter") AND (predict or prediction) 69 results

Search Terms – Detection of AF in Stroke Population

<u>Ovid MEDLINE(R) and Epub Ahead of Print, In-Process, In-Data-Review and Other Non-Indexed Citations, Daily and Versions <1946 to September 20, 2022></u>

1 (af or atrial fibrillation or atrial flutter*).ti,ab,kw. 105677
2 atrial fibrillation/ or atrial flutter/ 70394
3 detect*.ti,ab,kw. 2667685
4 (stroke or cerebral infarction or cerebrovascular accident or isch* stroke or cerebrovascular accident or brain isch* or isch* attack or cerebral isch* or thromboemb* or apoplex*).ti,ab,kw. 396482
5 Stroke/ or Embolic Stroke/ or Ischemic Stroke/ or Thrombotic Stroke/ or Stroke, Lacunar/ 128922
6 exp Stroke/ 163665
7 (machine learning or deep learning or neural network* or ai or artificial intelligence or decision tree or gradient boost* or XGBoost or Catboost or Bayes or SVM or support vector machine or random forest).ti,ab,kw. 228579
8 artificial intelligence/ or machine learning/ or deep learning/ or supervised machine learning/ 71951
9 1 or 2 118611
10 4 or 5 or 6 426807
11 7 or 8 247461
12 3 and 9 and 10 and 11
102 results

<u>Embase <1974 to 2022 September 20></u>

1 (af or atrial fibrillation or atrial flutter*).ti,ab,kw. 185112
2 atrial fibrillation/ or atrial flutter/ 102049
3 detect*.ti,ab,kw. 3391606
4 (stroke or cerebral infarction or cerebrovascular accident or isch* stroke or cerebrovascular accident or brain isch* or isch* attack or cerebral isch* or thromboemb* or apoplex*).ti,ab,kw. 618454
5 exp Stroke/ 277025
6 cerebrovascular accident/ or cardioembolic stroke/ or ischemic stroke/ or lacunar stroke/ 272014
7 (machine learning or deep learning or neural network* or ai or artificial intelligence or decision tree or gradient boost* or XGBoost or Catboost or Bayes or SVM or support vector machine or random forest).ti,ab,kw. 279848
8 artificial intelligence/ or machine learning/ or deep learning/ or supervised machine learning/ 130437
9 1 or 2 212881
10 4 or 5 or 6 686227
11 7 or 8 307847
12 3 and 9 and 10 and 11
166 results

Web of Science
(((TS=(af or atrial fibrillation or atrial flutter*)) AND TS=(detect*)) AND TS=(stroke or cerebral infarction or cerebrovascular accident or isch* stroke or cerebrovascular accident or brain isch* or isch* attack or cerebral isch* or thromboemb* or apoplex*)) AND TS=(machine learning or deep learning or neural network* or ai or artificial intelligence or decision tree or gradient boost* or XGBoost or Catboost or Bayes or SVM or support vector machine or random forest) 215 results

WHO ICTRP

("Atrial Fibrillation" OR "Atrial flutter") AND (detect OR detection) AND (stroke OR "cerebral ischemia" OR "cerebral ischemia" OR “cerebral infarction” OR “brain ischemia” OR stroke OR “cerebrovascular accident”) 45 results

Clinicaltrials.gov
("Atrial Fibrillation" OR "Atrial flutter") AND (detect OR detection) AND (stroke OR "cerebral ischemia" OR "cerebral ischemia" OR “cerebral infarction” OR “brain ischemia” OR stroke OR “cerebrovascular accident”) 53 results

Table S1. PRISMA checklist.
Preferred Reporting Items for Systematic reviews and Meta-Analyses extension for Scoping Reviews (PRISMA-ScR) Checklist

SECTION	ITEM	PRISMA-ScR CHECKLIST ITEM	REPORTED ON PAGE #
**TITLE**			
Title	1	Identify the report as a scoping review.	P1
**ABSTRACT**			
Structured summary	2	Provide a structured summary that includes (as applicable): background, objectives, eligibility criteria, sources of evidence, charting methods, results, and conclusions that relate to the review questions and objectives.	P1
**INTRODUCTION**			
Rationale	3	Describe the rationale for the review in the context of what is already known. Explain why the review questions/objectives lend themselves to a scoping review approach.	P1-P2
Objectives	4	Provide an explicit statement of the questions and objectives being addressed with reference to their key elements (e.g., population or participants, concepts, and context) or other relevant key elements used to conceptualize the review questions and/or objectives.	P2-3
**METHODS**			
Protocol and registration	5	Indicate whether a review protocol exists; state if and where it can be accessed (e.g., a Web address); and if available, provide registration information, including the registration number.	N/A
Eligibility criteria	6	Specify characteristics of the sources of evidence used as eligibility criteria (e.g., years considered, language, and publication status), and provide a rationale.	P3
Information sources*	7	Describe all information sources in the search (e.g., databases with dates of coverage and contact with authors to identify additional sources), as well as the date the most recent search was executed.	P3
Search	8	Present the full electronic search strategy for at least 1 database, including any limits used, such that it could be repeated.	P3, Supplement P2-4
Selection of sources of evidence†	9	State the process for selecting sources of evidence (i.e., screening and eligibility) included in the scoping review.	P2,3
Data charting process‡	10	Describe the methods of charting data from the included sources of evidence (e.g., calibrated forms or forms that have been tested by the team before their use, and whether data charting was done independently or in duplicate) and any processes for obtaining and confirming data from investigators.	P3,4
Data items	11	List and define all variables for which data were sought and any assumptions and simplifications made.	P4
Critical appraisal of individual sources of evidence§	12	If done, provide a rationale for conducting a critical appraisal of included sources of evidence; describe the methods used and how this information was used in any data synthesis (if appropriate).	N/A
Synthesis of results	13	Describe the methods of handling and summarizing the data that were charted.	N/A
**RESULTS**			

SECTION	ITEM	PRISMA-ScR CHECKLIST ITEM	REPORTED ON PAGE #
Selection of sources of evidence	14	Give numbers of sources of evidence screened, assessed for eligibility, and included in the review, with reasons for exclusions at each stage, ideally using a flow diagram.	P4, P8 Figure S1, S2
Characteristics of sources of evidence	15	For each source of evidence, present characteristics for which data were charted and provide the citations.	P4, P8, Table S3, S7
Critical appraisal within sources of evidence	16	If done, present data on critical appraisal of included sources of evidence (see item 12).	N/A
Results of individual sources of evidence	17	For each included source of evidence, present the relevant data that were charted that relate to the review questions and objectives.	P4-P9
Synthesis of results	18	Summarize and/or present the charting results as they relate to the review questions and objectives.	P4-P9
**DISCUSSION**			
Summary of evidence	19	Summarize the main results (including an overview of concepts, themes, and types of evidence available), link to the review questions and objectives, and consider the relevance to key groups.	P9-11
Limitations	20	Discuss the limitations of the scoping review process.	P11
Conclusions	21	Provide a general interpretation of the results with respect to the review questions and objectives, as well as potential implications and/or next steps.	P11-12
**FUNDING**			
Funding	22	Describe sources of funding for the included sources of evidence, as well as sources of funding for the scoping review. Describe the role of the funders of the scoping review.	P12

JBI = Joanna Briggs Institute; PRISMA-ScR = Preferred Reporting Items for Systematic reviews and Meta-Analyses extension for Scoping Reviews.

* Where *sources of evidence* (see second footnote) are compiled from, such as bibliographic databases, social media platforms, and Web sites.

† A more inclusive/heterogeneous term used to account for the different types of evidence or data sources (e.g., quantitative and/or qualitative research, expert opinion, and policy documents) that may be eligible in a scoping review as opposed to only studies. This is not to be confused with *information sources* (see first footnote).

‡ The frameworks by Arksey and O'Malley (6) and Levac and colleagues (7) and the JBI guidance (4, 5) refer to the process of data extraction in a scoping review as data charting.

§ The process of systematically examining research evidence to assess its validity, results, and relevance before using it to inform a decision. This term is used for items 12 and 19 instead of "risk of bias" (which is more applicable to systematic reviews of interventions) to include and acknowledge the various sources of evidence that may be used in a scoping review (e.g., quantitative and/or qualitative research, expert opinion, and policy document).

From: Tricco AC, Lillie E, Zarin W, O'Brien KK, Colquhoun H, Levac D et al. PRISMA Extension for Scoping Reviews (PRISMAScR): Checklist and Explanation. Ann Intern Med. 2018;169:467–473. doi: 10.7326/M18-0850.

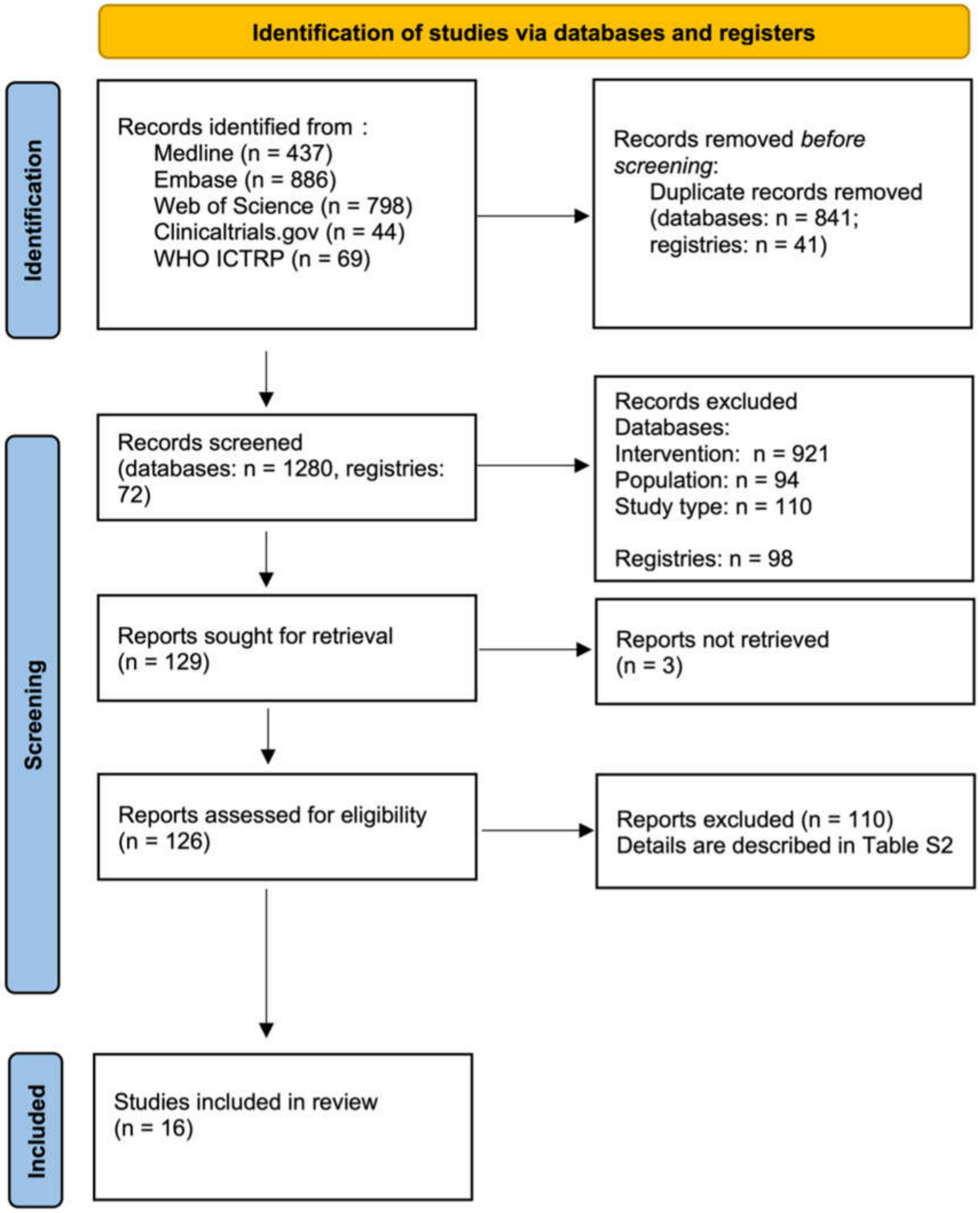

Figure S1. PRISMA Flow Diagram of Studies Describing Prediction of Incident Atrial Fibrillation in a Population without Prior AF.

Table S2. Excluded Studies for Prediction of Incident Atrial Fibrillation in Population without Prior AF.

Study	Reason for exclusion
Acharya et al. 2008 [1]	Study was about detection, rather than prediction of atrial fibrillation
Ahlberg et al. 2018 [2]	Not enough information for 2x2 table
Ahsanuzzaman et al. 2020 [3]	Not enough information for 2x2 table
Anetta et al. 2022 [4]	Patient cohort was entirely cardiology-based patients
Arvaneh et al. 2009 [5]	Not observational cohort study; evaluated on MIT-BIH dataset
Attia et al. 2019 [6]	Study included patients with known history of AF
Baek et al. 2021 [7]	Study was about detection, rather than prediction of atrial fibrillation
Barbieri et al. 2022 [8]	Study predicted cardiovascular events in general rather than atrial fibrillation or flutter
Bertsimas et al. 2021 [9]	Study was about detection, rather than prediction of atrial fibrillation
Boon et al. 2018 [10]	Not observational cohort study; evaluated on Physionet dataset
Boon et al. 2016 [11]	Not observational cohort study; evaluated on Physionet dataset
Bratt et al. 2019 [12]	Study included patients with known history of AF
Bui et al. 2020 [13]	Text not accessible
Bundy et al. 2020 [14]	Does not use machine learning for prediction
Cho et al. 2018 [15]	Model trained and evaluated only on patients with AF
Chua et al. 2019 [16]	Study included patients with known history of AF
De Giovanni et al. 2017 [17]	Study included patients with known history of AF
Castro et al. 2021 [18]	Study included patients with known history of AF
Chen et al. 2020 [19]	Study included patients with known history of AF

De Jong et al. 2021 [20]	Does not use machine learning-based prediction models
Derevitskii et al. 2021 [21]	Includes patients with underlying medical condition
Ebrahimzadeh et al. 2018 [22]	Study included patients with known history of AF
Egmont-Petersen et al. 1999 [23]	Includes patients with underlying medical condition (treated with cardiac surgery)
Erdenebeyer et al. 2009 [24]	Study included patients with known history of AF
Faulx et al. 2021 [25]	Study type was not research article
Fernandez-Fernandez et al. 2019 [26]	Study type was not research article
Filos et al. 2017 [27]	Study included patients with known history of AF
ElMoaqet et al. 2017 [28]	Study was performed on animals, not humans
Firyulina et al. 2020 [29]	Study classified, rather than predicted, atrial fibrillation
Fujita et al. 2019 [30]	Study classified, rather than predicted, atrial fibrillation
Gilon et al. 2020 [31]	Study excluded patients on pacemakers, but did not explicitly exclude patients with known history of AF
Gonzalez-Cordero et al. 2021 [32]	Study included patients with known history of AF
Gregoire et al. 2020 [33]	Study does not include information about patient cohort
Gregoire et al. 2022 [34]	Study included patients with known history of AF
Gregoire et al. 2020 [35]	Study does not include information about patient cohort
Gregoire et al. 2021 [36]	Study does not include information about patient cohort
Gregoire et al. 2019 [37]	Study does not include information about patient cohort
Grout et al. 2021 [38]	Model in study was based on logistic regression
Guo et al. 2021 [39]	Study does not include information about patient cohort
Hayn et al. 2007 [40]	Text not accessible

Henry et al. 2022 [41]	Study does not exclude patients with known history of AF
Heo et al. 2021 [42]	Study is on a stroke cohort
Hill et al. 2018 [43]	Preliminary study for Hill et al. 2019 [44]<br>Used LASSO logistic regression, neural networks, random forests, and SVM used to predict incidence of AF. Logistic regression, neural networks, random forests, and SVM had AUC of 0.811, 0.811, 0.812, and 0.818, respectively and all models outperformed Cox regression.
Hill et al. 2020 [45]	Study protocol
Hirota et al. 2020 [46]	Model in study was based on logistic regression
Hong et al. 2020 [47]	Study included patients with known history of AF
Hsieh et al. 2022 [48]	Study included patients with known history of AF
Hu et al. 2019 [49]	Study included patients with known history of AF
Jalali et al. 2020 [50]	Study included patients with known history of AF
Jyothi et al. 2022 [51]	Text not accessible
Karnik et al. 2012 [52]	Study included patients with known history of AF
Kastruiwale et al. 2021 [53]	Study included patients with known history of AF
Kawakami et al. 2022 [54]	Study included patients with known history of AF
Kim et al. 2019 [55]	Preliminary study on same dataset as Kim et al. 2020 [56]
Kim et al. 2022 [57]	Study was performed on patients with underlying conduction problems
Kordik et al. 2008 [58]	Study classified, rather than predicted, atrial fibrillation
Krstacic et al. 2001 [59]	Not observational cohort study
Kwon et al. 2022 [60]	Study included patients with known history of AF
Lehtonen et al. 2019 [61]	Not primary research article
Li et al. 2021 [62]	Not observational cohort study

Liu et al. 2021 [63]	Study was performed on inpatients at a respiratory ward and not on a general population
Liu et al. 2021 [64]	Study was performed on patients undergoing atrial appendage removal
Loring et al. 2020 [65]	Study included patients with known history of AF
Lou et al. 2022 [66]	Study did not exclude patients with known history of AF
Maghawry et al. 2021 [67]	Not observational cohort study
McMillan et al. 2012 [68]	Study did not exclude patients with known history of AF
Melzi et al. 2021 [69]	Study did not exclude patients with known history of AF
Mendez et al. 2022 [70]	Not observational cohort study
Mohebbi et al. 2011 [71]	Not observational cohort study
Mohebbi et al. 2012 [72]	Not observational cohort study
Mohebbi et al. 2011 [73]	Not observational cohort study
Mroueh et al. 2019 [74]	Not observational cohort study
Nadarajah et al. 2021 [75]	Study protocol
Okutucu et al. 2017 [76]	Study was on patients with history of AF
Parsi et al. 2021 [77]	Not observational cohort study
Ponomartseva et al. 2021 [78]	Study was performed on patients with underlying hyperthyroidism
Pourbabaee et al. 2008 [79]	Not observational cohort study
Raghunath et al. 2019 [80]	Preliminary study on same dataset as Raghunath et al. 2021 [81]
Ramirez et al. 2021 [82]	Does not give AUC or information to create a 2x2 table
Ravens et al. 2015 [83]	Study included patients with known history of AF
Ravish et al. 2014 [84]	Not observational cohort study
Safabakhsh et al. 2020 [85]	Not observational cohort study
Shen et al. 2020 [86]	Not observational cohort study
Sovilj et al. 2011 [87]	Study included patients with known history of AF
Sun et al. 2020 [88]	Not observational cohort study
Surucu et al. 2021 [89]	Not observational cohort study
Suzuki et al. 2022 [90]	Study included patients with known history of AF
Szep et al. 2019 [91]	Not observational cohort study
Tabassum et al. 2016 [92]	Not observational cohort study
Taggar et al. 2020 [93]	Not primary research article

Talukdar et al. 2018 [94]	Primary outcome is not AF
Tieleman et al. 2019 [95]	Article not in English
Tse et al. 2020 [96]	Patients had underlying mitral valve stenosis
Tzou et al. 2021 [97]	Study included patients with known history of AF
Wang et al. 2021 [98]	Not observational cohort study
Wang et al. 2022 [99]	Primary outcome was not incidence of AF
Wang et al. 2022 [100]	Not observational cohort study
Wehbe et al. 2020 [101]	Not primary research article
Wu et al. 2021 [102]	Not observational cohort study
Xie et al. 2021 [103]	Not primary research article
Xin et al. 2017 [104]	Not observational cohort study
Yang et al. 2022 [105]	Study included patients with known history of AF
Ye et al. 2021 [106]	Study was about classification, rather than prediction of atrial fibrillation
Zhang et al. 2020 [107]	Not observational cohort study
Zhang et al. 2022 [108]	Not observational cohort study
NCT02307032	No results available
NCT03130985	All patients had undergone cardiac surgery
NCT03937089	No results available
NCT04045639	Data published in Hill et al. [44]
NCT03357926	No results available
NCT01171040	Did not use machine learning
NCT01405209	No results available
NCT04655443	Did not use machine learning
NCT05045742	No results available
ISRCTN17993837	No results available
ACTRN12620000929909	No results available
JPRN-UMIN000031719	Did not use machine learning
JPRN-UMIN000020676	No results available
JPRN-UMIN000007911	No results available
JPRN-UMIN000005419	Did not use machine learning
JPRN-UMIN000004536	No results available
ISRCTN62172102	No results available

Table S3. Population Characteristics of Studies for Prediction of Atrial Fibrillation in General Population

Study	Country	Study population (% prevalence)	Eligibility criteria	Follow-up period (Study dates)	Study aims
Ahmad et al. 2020[109]	Lebanon	n = 232 (ratio not provided)	Patients with no history of stroke or AF	3 months (not provided)	Use left atrium strain to predict AF
Ambale-Venkatesh et al. 2017[110]	United States	n = 6,814 (4.7%)	Free of cardiovascular disease at enrolment	12 years (Baseline between 2000-2002)	Characterize cardiovascular risk, predict outcomes, and identify biomarkers
Christopoulos et al. 2020[111]	United States	n = 1936 (17.2%)	Documented to be free of AF and no AF at baseline. At least one sinus EKG within 2 years before baseline	10 years (Baseline 2004 and 2012)	Relationship of AI model with clinical risk scores and performance of AI model
Hill et al. 2019[44]	United Kingdom	n = 2,994,287 (3.2%)	>30 years of age and no history of AF in 5 years prior to study period	11 years (Study period Jan 2006-Dec 2016)	Develop clinically applicable risk prediction model to identify between baseline and time-varying factors and identification of AF
Hirota et al. 2021[112]	Japan	n = 11,732 (0.8%)	No history of AF and sinus EKG at and within 30 days of initial visit	8 years (2010-2018)	Assess predictive capability of parameters obtained from EKG
Hu et al. 2019[113]	Taiwan	n = 682,237 (2.1%)	>18 years of age and no diagnosis of AF before initial visit, with complete medical information	13 years (2000-2013)	Develop AI model to predict AF for an Asian population
Joo et al. 2020[114]	South Korea	n = 297,875 (3.0% at 2 years;12.3% at 10 years)	No history of AF, coronary artery disease, heart failure, hemorrhagic stroke, ischemic stroke	2 years/10 years (Baseline 2003)	Develop AI model to predict AF for an Asian population, compare short- and long-term risk, evaluate physician bias
Kaminski et al. 2022[115]	United States	n = 1403 (3.1%)	No history of AF with sinus 12-lead EKG performed at initial visit and not used for ECG-AI model development	1 year (2017-2020)	Validate performance of ECG-AI on external cohort

Khurshid et al. 2022[116]	United States, United Kingdom (validation cohort)	C3PO cohort: n = 87899 (12.9 per 1000 person years)<br>UK biobank cohort n = 41,033 (4.2 per 1000 person years)	Internal validation: 18-90 years of age without history of AF<br>External validation: 40-69 years of age without history of AF	5 years (2000-2019; C3PO)<br>2 years (2006-2010; UK Biobank)	Develop AI model to predict time to AF
Kim et al. 2020[56]	South Korea	n = 432,587 (1.4%)	>18 years of age without diagnosis of AF or valvular AF or change in ZIP code or missing data	4 years (2009-2013)	Find risk factors for incident AF using machine learning and regression methods
Kim et al. 2020[117]	South Korea	n= 258,896 (not reported)	Middle-aged individuals free of CVD at baseline	3 years (2009-2013)	Compare the contribution of different data types in prediction of AF
Lip et al. 2022[118]	United States	n = 617,483 (0.49 per person years)	Medicaid patients aged between 18 to 90 years with at least 30 months enrolment and at least 24 months without diagnosis of AF at baseline	5 years (2016-2021)	Report incidence of AF in Medicaid population and use AI models to predict AF incidence and complications accounting for demographic groups
Raghunath et al. 2021[81]	United States	n = 287,593 (3.5%)	>18 years of age without history of AF with ECG available without significant artefact	1 year (1996 -2020)	Use AI model to predict new-onset AF in patients without history of AF
Schnabel et al. 2023[119]	Germany	n = 1,476,391 (6.7%)	>18 years of age without history of AF	2 years (2013-2015)	Identify set of routinely available AF- and stroke-related AF risk predictors and integrate into AI model
Sekelj et al. 2021[120]	United Kingdom	n = 604,135 (3.9%)	>30 years of age and no history of AF in 5 years prior to study period	16 years (2001-2016)	Externally validate model in Hill et al. 2019[17]
Tiwari et al. 2020[121]	United States	n = 2,252,219 (1.2%)	Patients without a history of AF	7 years (incident AF predicted multiple 6-month intervals; 2011-2018)	Develop and test AI model for AF prediction

Table S4. Characteristics of Models for Prediction of Atrial Fibrillation in the General Population.

Study	Input data	Data source/ Data curated for approved access?	Model	Model architecture*	Validation	Results	Model interpretation	Code or model available	Model currently available for clinical use?	Reported handling of sparse data
Ahmad et al. 2020[109]	Clinical features, strain value	Retrospective Local EHR/no	New	Adaboost	Unclear	AUC: 0.82	No	Neither	No	No
Ambale-Venkatesh et al. 2017[110]	ECG parameters, clinical features, biomarker data	Prospective Database (MESA cohort)/yes	New	Random forest	Internal	AUC: 0.86	Yes	Neither	No	Yes (adaptive imputation)
Christopoulos et al. 2020[111]	ECG trace, clinical data	Prospective Multiple local EHRs (MCSA cohort)/ yes	ECG-AI[29]	CNN + Cox regression	Internal[a]	AUC: 0.72	No	Neither	No	No
Hill et al. 2019[44]	Clinical features	Retrospective database/yes	New	Neural network	External	AUC: 0.83	Yes	Neither	No	No
Hirota et al. 2021[112]	ECG parameters	Retrospective database (Shinken database cohort)/no	New	Random forest	Internal	AUC: 0.99	Yes	Neither	No	No
Hu et al. 2019[113]	Clinical features	Retrospective database (Taiwan National Health Insurance Database)/yes	New	Random forest	Internal; Validation on larger cohort (incl. study cohort) AUC: 0.850.	AUC: 0.95	Yes	Neither	No	No

Joo et al. 2020[114]	Clinical features	Retrospective (Korean National Health Insurance Database)/yes	New	Neural network*, random forest, lightGBM*	Internal	2 years AUC: 0.78<br><br>10 years AUC: 0.75	Yes	Neither	No	No
Kaminski et al. 2022[115]	ECG trace, clinical data	Multiple retrospective local EHR/no	ECG-AI[29]	CNN + Cox regression	Internal[a]	AUC: 0.74	No	Neither	No	No
Khurshid et al. 2022[116]	ECG traces, clinical features	Retrospective databases/yes	New	CNN + Cox regression	External	Internal validation – AUC:0.84 External validation – average AUC: 0.76	Yes	Neither	No	Yes (but not in training)
Kim et al. 2020[117]	Clinical features	Retrospectived atabase (Korean National Health Insurance database)/yes	New	SVM, decision tree, random forest*, Naïve Bayes, deep neural network, XGBoost	Internal	AUC: 0.95	Yes	Neither	No	No (patients with missing data excluded)
Kim et al. 2020[117]	Clinical features	Retrospective database (Korean National Health Insurance database)/yes	New	Neural Network	Internal	AUC: 0.69	No	Neither	No	No
Lip et al. 2022[118]	Clinical features	Retrospective database/yes	New	Neural network	Internal	AUC: 0.84	No	Neither	No	No

Raghunath et al. 2021[81]	ECG traces, clinical features	Multiple retrospective local EHRs/no	New	CNN	External (simulated by splitting data based on hospital)	AUC: 0.84 Simulated external validation AUC: 0.85	No	Neither	No	No
Schnabel et al. 2023[119]	Clinical features	Retrospective database/proprietary	New	Gradient boosted trees	Temporal and external	AUC: 0.82 (temporal validation), 0.76 (external validation)	Yes	Neither	No	No
Sekelj et al. 2021[120]	Clinical features	Retrospective database (DISCOVER cohort)/yes	Hill et al. 2019 [17]	Neural network	External	AUC: 0.87	No	Neither	No	No
Tiwari et al. 2020[121]	Clinical features	Multiple retrospective local EHRs/no	New	Naïve Bayes, Logistic regression, Random forest, Gradient boosted trees, Neural network*	Internal	AUC: 0.80	No	Neither	No	No

[a]Model proposed in Attia et al. 2019 [6] (not included in this study) was originally developed for detection rather than prediction of atrial fibrillation.
*Best-performing model if multiple models were tested

Table S5. Features Used in Final Model Training.

Features \ Studies	Ahmad et al. 2020 [109]	Ambale-Venkatesh et	Christopoulos et al. 2020	Hill et al. 2019 [44]	Hirota et al. 2021 [112]	Hu et al. 2019 [113]	Joo et al. 2020 [114]	Kaminski et al. 2022 [115]	Khurshid et al. 2022 [116]	Kim et al. 2020 [56]	Kim et al. 2020 [117],*	Lip et al. 2022 [118]	Rabinstein et al. 2021 [119]	Raghunath et al. 2021 [81]	Reinke et al. 2018 [120]	Schnabel et al. 2023 [121]	Sekelj et al. 2021 [122]	Shan et al. 2014 [123]	Tiwari et al. 2020 [124]
Age	X	X	X	X		X	X		X	X	X	X		X		X	X		
Sex		X		X		X	X		X		X	X		X		X			
Race		X	X	X					X										
BMI/obesity	X	X	X	X			X		X	X	X	X					X		X
Blood pressure		X	X	X			X		X	X	X						X		X
Heart rate		X																	
Hypoxemia																			X
Creatinine		X																	
Blood sugar		X								X									
GGT							X			X									
HMG							X			X									
Gross proteinuria							X												
Hypokalemia																			X
SAST							X			X									
SALT							X			X									
Cholesterol/triglycerides		X				X	x			X									X
Diabetes		X	X	X		X	X	X	X	X		X							X
GERD																			X
Diarrhea																			X
Smoking		X	X	X			X		X	X	X								
Exercise		X					X												
Alcohol		X					X			X									
Pregnancy																			X
Family history of CVD		X									X								X
Peripheral pain																			X
Nausea/fever/dizziness																			X
Headache																			X
Infection/vaccination																			x

Allergic rhinitis																			X
Emphysema		X																	
Asthma		X										X							X
COPD						X				X		X							X
Acute bronchitis																			X
Sleep apnea						X						X							X
Rheumatologic disease						X													
Thyroid disease												X							X
Arthritis		X										X							X
Spondylosis												X							
Lower limb ulcer																X			
Cancer		X				X	X												
Constipation																			X
Cognitive impairment/anxiety												X							X
Liver disease		X										X							
Gout						X													
CKD/ESRD						X				X	X	X				X			
Dysuria																			X
Anemia						X													X
Major bleeding												X							
Hypertension (chronic)				X		X	X	X		X		X				X			X
Heart failure	X		X	X		X		X	X	X		X				X			
CAD/MI	X		X	X		X			X	X		X				X			X
CVA/TIA						X		X		X		X				X			
Peripheral Artery Disease												X							
Congenital Heart Disease				X															
Valvular Heart Disease	X											X				X			
Pulmonary Heart Disease																X			
Murmur Presence	X																		
Other arrythmias/Tachycardia																X			X
Cardiovascular Drugs	X	X	X	X			X		X	X	X					X	X		X
Other drugs																			X
Site		X																	

CHA_2DS_2-VASc						X		X				X							
MRI measurements		X																	
Atherosclerosis (CT/US)		X																	
Echo parameters	X	X																	
ECG traces			X					X	X				X	X					
ECG parameters		X			X										X				
PPG parameters																		X	
Blood Biomarkers**		X																	
Socioeconomic		X								X	X	X							

BMI: Body Mass Index; GGT: Gamma-Glutamyl Transferase; HMG: Hemoglobin; SAST: Serum Aspartate Aminotransferase; SALT: Serum Alanine Aminotransferase; GERD: Gastro-esophageal Reflux Disease; CVD: Cardiovascular Disease; COPD: Chronic Obstructive Pulmonary Disease; CKD: Chronic Kidney Disease; ESRD: End-stage Renal Disease; CAD: Coronary Artery Disease; MI: Myocardial Infarction; CVA: Cerebrovascular Accident; TIA: Transient Ischemic Attack; MRI: Magnetic Resonance Imaging; CT: Computed Tomography; US: Ultrasound; ECG: Electrocardiogram; PPG: Photoplethysmography

*Not clear as factors not explicitly enumerated and had to be inferred.

** Interleukin-2 soluble receptor, plasmin–antiplasmin complex, d-dimer, Factor VIII, NT-proBNP (N-Terminal Pro-B-Type Natriuretic Peptide), cardiac troponin-T, C-reactive protein, interleukin-6, fibrinogen, homocysteine, tissue necrosis factor-α soluble receptor

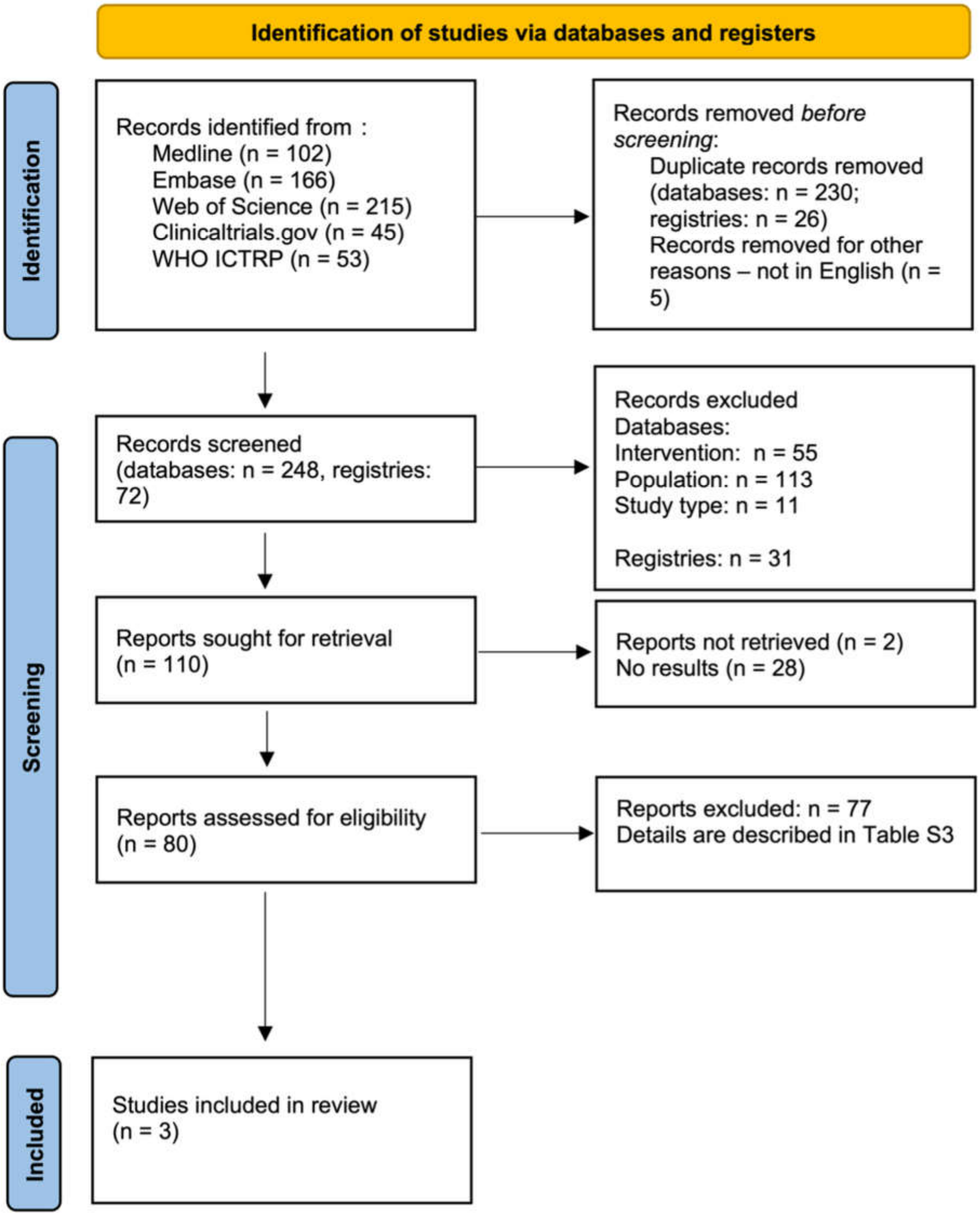

Figure S2. PRISMA Flow Diagram of Studies Describing Detection of Atrial Fibrillation in a Stroke Population.

Table S6. Excluded Studies for Detection of Atrial Fibrillation after Stroke.

Study	Reason for exclusion
Abdul-Kadir et al. [125]	Not stroke cohort
Baek et al. [7]	Not stroke cohort
Bahrami et al. [126]	Not stroke cohort
Ben Itzhak et al. [127]	Not stroke cohort
Chantercrob et al. [128]	Not stroke cohort
Couceiro et al. [129]	Not stroke cohort
Duverney et al. [130]	Not stroke cohort
Eerikainen et al. [131]	Not stroke cohort
Fan et al. [132]	Not stroke cohort
Faust et al. [133]	Not stroke cohort
Helfenbein et al. [134]	Not stroke cohort
Ivanovic et al. [135]	Not stroke cohort
Jia et al. [136]	Not stroke cohort
Kennedy et al. [137]	Not stroke cohort
Kim et al. [138]	Not stroke cohort
Krol-Jozaga et al. [139]	Not stroke cohort
Lee et al. [140]	Not stroke cohort
Lee et al. 2017 [141]	Not stroke cohort
Lee et al. 2020 [142]	Not stroke cohort
Lei et al. 2021 [143]	Not stroke cohort
Leutheuser et al. 2015 [144]	Not stroke cohort
Liaqat et al. 2020 [145]	Not stroke cohort
Liu et al. 2022 [146]	Not stroke cohort
Medic et al. 2021 [147]	Does not measure outcomes required for this review
Mittal et al. 2021 [148]	Not stroke cohort
Mousavi et al. 2020 [149]	Not stroke cohort
Nguyen et al. 2019 [150]	Not stroke cohort
Nuryani et al. 2015 [151]	Not stroke cohort
Park et al. 2009 [152]	Not stroke cohort
Pereira et al. 2019 [153]	Not stroke cohort
Pham et al. 2021 [154]	Not stroke cohort
Piccini et al. 2021 [155]	Does not measure outcomes required for this review
Piorkowski et al. 2019 [156]	Not stroke cohort
Pokaprakarn et al. 2022 [157]	Not stroke cohort
Pollock et al. 2020 [158]	Not stroke cohort
Qayyum et al. 2018 [159]	Not stroke cohort
Quartieri et al. 2019 [160]	Not stroke cohort
Rahul et al. 2022 [161]	Not stroke cohort
Rosa et al. 2021 [162]	Not stroke cohort
Sahu et al. 2022 [163]	Not stroke cohort
Sandberg et al. 2021 [164]	Not stroke cohort

Sasaki et al. 2019 [165]	Not stroke cohort
Schäck et al. 2017 [166]	Not stroke cohort
Sideshwar et al. 2021 [167]	Not stroke cohort
Sims et al. 2021 [168]	Does not measure outcomes required for this review
Sun et al. 2022 [169]	Not stroke cohort
Tadi et al. 2019 [170]	Not stroke cohort
Taniguchi et al. 2021 [171]	Not stroke cohort
Tison et al. 2018 [172]	Not stroke cohort
Ukil et al. 2022 [173]	Not stroke cohort
Wang et al. 2022 [100]	Does not measure outcomes required for this review
Wong et al. 2022 [174]	Study type is a protocol rather than a research article
Xia et al. 2018 [175]	Not stroke cohort
Yao et al. 2017 [176]	Not stroke cohort
Yokokawa et al. 2020 [177]	Study included non-stroke patients
Yu et al. 2020 [178]	Not stroke cohort
Yue et al. 2019 [179]	Not stroke cohort
Zalabarria et al. 2020 [180]	Not stroke cohort
Zhu et al. 2022 [181]	Not stroke cohort
Clinical trial NCT0132554 Baturova et al. 2016 [182]	Does not use machine learning
Clinical trial NCT01858779 Kallmunzer et al. 2015 [183]	Does not use machine learning
Clinical trial NCT02725944 Skrebelyte-Strøm et al. 2022 [184]	Does not use machine learning
Clinical trial NCT02725944 Haeusler et al. 2016 [185]	Does not use machine learning
Clinical trial NCT02204267 Haeusler et al. 2021 [186]	Does not use machine learning
Clinical trial NCT02261766 Poulsen et al. 2016 [187]	Does not use machine learning
Clinical trial NCT02578979 Huang et al. 2020 [188]	Does not use machine learning
Clinical trial NCT00279981 Glotzer et al. 2009 [189]	Not stroke cohort
Clinical trial NCT01855035 Wachter et al. 2017 [190]	Does not use machine learning
Clinical trial NCT02428140 Buck et al. 2021 [191]	Does not use machine learning
Clinical trial NCT02270112 Brasier et al. 2019 [192]	Not stroke cohort

Clinical trial NCT0372160	Does not use machine learning
NCT0350733	Not stroke cohort
Pereira et al. 2020 [193]	Primary outcome was to classify quality of signal, rather than its nature
Schnabel et al. 2022 [121]	Study is about prediction rather than detection of stroke
Sung et al. 2022 [194]	Study is about prediction rather than detection of stroke
Shan et al. 2016 [123]	Does not exclude patients with history of AF

Table S7. Population Characteristics of Studies for Detection of Atrial Fibrillation in a Stroke Population.

Study	Country	Study population (% prevalence)	Eligibility criteria	Follow-up period (Study dates)	Study aims
Rabinstein et al. 2021[119]	United States	n = 265 (4.1%)	Patients admitted for embolic stroke of unknown cause (ESUS)	Up to 30 days (2018-2019)	Test whether AI model can discriminate patients with embolic stroke of unknown cause and whether predicted AF probability is associated with AF identified through ambulatory monitoring
Reinke et al. 2018[120]	Germany	n = 105 (18%)	Patients admitted with cryptogenic stroke	20 months (2013-2014)	Assess ability of AI software to detect AF and develop cost-effective strategy to optimize detection of AF
Shan et al. 2014[123]	Taiwan	n = 468 (not reported)	Patients in stroke unit	Not reported (2012-2014)	Propose a photoplethysmography-based AI detection model of AF

References

1. Acharya, U.R.; Sankaranarayanan, M.; Nayak, J.; Xiang, C.; Tamura, T. Automatic identification of cardiac health using modeling techniques: A comparative study. *INFORMATION SCIENCES* **2008**, *178*, 4571-4582, doi:10.1016/j.ins.2008.08.006.
2. Ahlberg, G.; Wang, L.; Roselli, C.; Chaffin, M.; Hoan Choi, S.; Ghouse, J.; Hanso, S.; Svendsen, J.H.; Olesen, M.S.; Lubitz, S.; et al. The genetic risk and interactions in atrial fibrillation, evidence from 335,070 UK biobank participants. *European Heart Journal* **2018**, *39*, 870, doi:https://dx.doi.org/10.1093/eurheartj/ehy563.4346.
3. Ahsanuzzaman, S.M.; Ahmed, T.; Rahman, M.A.; Ieee. Low Cost, Portable ECG Monitoring and Alarming System Based on Deep Learning. In Proceedings of the 2020 IEEE REGION 10 SYMPOSIUM (TENSYMP) - TECHNOLOGY FOR IMPACTFUL SUSTAINABLE DEVELOPMENT, 2020; pp. 316-319.
4. Anetta, K.; Horak, A.; Wojakowski, W.; Wita, K.; Jadczyk, T. Deep Learning Analysis of Polish Electronic Health Records for Diagnosis Prediction in Patients with Cardiovascular Diseases. *Journal of Personalized Medicine* **2022**, *12*, 869, doi:https://dx.doi.org/10.3390/jpm12060869.
5. Arvaneh, M.; Ahmadi, H.; Azemi, A.; Shajiee, M.; Dastgheib, Z.S.; Ieee. Prediction of Paroxysmal Atrial Fibrillation by Dynamic Modeling of the PR Interval of ECG. In Proceedings of the 2009 INTERNATIONAL CONFERENCE ON BIOMEDICAL AND PHARMACEUTICAL ENGINEERING, 2009; pp. 255-+.
6. Attia, Z.I.; Noseworthy, P.A.; Lopez-Jimenez, F.; Asirvatham, S.J.; Deshmukh, A.J.; Gersh, B.J.; Carter, R.E.; Yao, X.; Rabinstein, A.A.; Erickson, B.J.; et al. An artificial intelligence-enabled ECG algorithm for the identification of patients with atrial fibrillation during sinus rhythm: a retrospective analysis of outcome prediction. *Lancet (London, England)* **2019**, *394*, 861-867, doi:https://dx.doi.org/10.1016/S0140-6736(19)31721-0.
7. Baek, Y.S.; Lee, S.C.; Choi, W.; Kim, D.H. A new deep learning algorithm of 12-lead electrocardiogram for identifying atrial fibrillation during sinus rhythm. *SCIENTIFIC REPORTS* **2021**, *11*, doi:10.1038/s41598-021-92172-5.
8. Barbieri, S.; Mehta, S.; Wu, B.; Bharat, C.; Poppe, K.; Jorm, L.; Jackson, R. Predicting cardiovascular risk from national administrative databases using a combined survival analysis and deep learning approach. *International Journal of Epidemiology* **2022**, *51*, 931-944, doi:https://dx.doi.org/10.1093/ije/dyab258.
9. Bertsimas, D.; Mingardi, L.; Stellato, B. Machine Learning for Real-Time Heart Disease Prediction. *IEEE Journal of Biomedical and Health Informatics* **2021**, *25*, 3627-3637, doi:https://dx.doi.org/10.1109/JBHI.2021.3066347.
10. Boon, K.H.; Khalil-Hani, M.; Malarvili, M.B. Paroxysmal atrial fibrillation prediction based on HRV analysis and non-dominated sorting genetic algorithm III. *Computer Methods and Programs in Biomedicine* **2018**, *153*, 171-184, doi:https://dx.doi.org/10.1016/j.cmpb.2017.10.012.
11. Boon, K.H.; Khalil-Hani, M.; Malarvili, M.B.; Sia, C.W. Paroxysmal atrial fibrillation prediction method with shorter HRV sequences. *Computer Methods and Programs in Biomedicine* **2016**, *134*, 187-196, doi:https://dx.doi.org/10.1016/j.cmpb.2016.07.016.
12. Bratt, A.; Guenther, Z.; Hahn, L.D.; Kadoch, M.; Adams, P.L.; Leung, A.N.C.; Guo, H.H. Left Atrial Volume as a Biomarker of Atrial Fibrillation at Routine Chest CT:

Deep Learning Approach. *Radiology. Cardiothoracic imaging* **2019**, *1*, e190057, doi:https://dx.doi.org/10.1148/ryct.2019190057.
13. Bui, C.T.; Huynh, P.K.; Phan, H.T.; Le, T.Q.; Toi, V.V. Developing Neural-fuzzy-based Unscented Kalman Filter Algorithm for Atrial Fibrillation Onset Prediction. In Proceedings of the 7TH INTERNATIONAL CONFERENCE ON THE DEVELOPMENT OF BIOMEDICAL ENGINEERING IN VIETNAM (BME7): TRANSLATIONAL HEALTH SCIENCE AND TECHNOLOGY FOR DEVELOPING COUNTRIES, 2020; pp. 119-125.
14. Bundy, J.D.; Heckbert, S.R.; Chen, L.Y.; Lloyd-Jones, D.M.; Greenland, P. Evaluation of Risk Prediction Models of Atrial Fibrillation (from the Multi-Ethnic Study of Atherosclerosis [MESA]). *American Journal of Cardiology* **2020**, *125*, 55-62, doi:https://dx.doi.org/10.1016/j.amjcard.2019.09.032.
15. Cho, J.; Kim, Y.; Lee, M. Prediction to Atrial Fibrillation Using Deep Convolutional Neural Networks. In Proceedings of the PREDICTIVE INTELLIGENCE IN MEDICINE, 2018; pp. 164-171.
16. Chua, W.; Purmah, Y.; Cardoso, V.R.; Gkoutos, G.V.; Tull, S.P.; Neculau, G.; Thomas, M.R.; Kotecha, D.; Lip, G.Y.H.; Kirchhof, P.; et al. Data-driven discovery and validation of circulating blood-based biomarkers associated with prevalent atrial fibrillation. *European heart journal* **2019**, *40*, 1268-1276, doi:https://dx.doi.org/10.1093/eurheartj/ehy815.
17. De Giovanni, E.; Aminifar, A.; Luca, A.; Yazdani, S.; Vesin, J.M.; Atienza, D.; Ieee. A Patient-Specific Methodology for Prediction of Paroxysmal Atrial Fibrillation Onset. In Proceedings of the 2017 COMPUTING IN CARDIOLOGY (CINC), 2017.
18. Castro, H.; Garcia-Racines, J.D.; Bernal-Norena, A. Methodology for the prediction of paroxysmal atrial fibrillation based on heart rate variability feature analysis. *Heliyon* **2021**, *7*, e08244, doi:https://dx.doi.org/10.1016/j.heliyon.2021.e08244.
19. Chen, Y.H.; Twinge, A.H.; Badawi, D.; Danavi, J.; McCauley, M.; Cain, A.E.; Ieee. ATRIAL FIBRILLATION RISK PREDICTION FROM ELECTROCARDIOGRAM AND RELATED HEALTH DATA WITH DEEP NEURAL NETWORK. In Proceedings of the 2020 IEEE INTERNATIONAL CONFERENCE ON ACOUSTICS, SPEECH, AND SIGNAL PROCESSING, 2020; pp. 1269-1273.
20. de Jong, V.M.T.; Moons, K.G.M.; Eijkemans, M.J.C.; Riley, R.D.; Debray, T.P.A. Developing more generalizable prediction models from pooled studies and large clustered data sets. *Statistics in Medicine* **2021**, *40*, 3533-3559, doi:https://dx.doi.org/10.1002/sim.8981.
21. Derevitskii, I.V.; Savitskaya, D.A.; Babenko, A.Y.; Kovalchuk, S.V. Hybrid predictive modelling: Thyrotoxic atrial fibrillation case. *JOURNAL OF COMPUTATIONAL SCIENCE* **2021**, *51*, doi:10.1016/j.jocs.2021.101365.
22. Ebrahimzadeh, E.; Kalantari, M.; Joulani, M.; Shahraki, R.S.; Fayaz, F.; Ahmadi, F. Prediction of paroxysmal Atrial Fibrillation: A machine learning based approach using combined feature vector and mixture of expert classification on HRV signal. *Computer methods and programs in biomedicine* **2018**, *165*, 53-67, doi:https://dx.doi.org/10.1016/j.cmpb.2018.07.014.
23. Egmont-Petersen, M.; Dassen, W.R.M.; Reiber, J.H.C. Sequential selection of discrete features for neural networks - A Bayesian approach to building a cascade. *PATTERN RECOGNITION LETTERS* **1999**, *20*, 1439-1448, doi:10.1016/S0167-8655(99)00112-9.

24. Erdenebayar, U.; Kim, H.; Park, J.-U.; Kang, D.; Lee, K.-J. Automatic Prediction of Atrial Fibrillation Based on Convolutional Neural Network Using a Short-term Normal Electrocardiogram Signal. *Journal of Korean medical science* **2019**, *34*, e64, doi:https://dx.doi.org/10.3346/jkms.2019.34.e64.
25. Faulx, M.D. Predicting incident atrial fibrillation using single channel nocturnal oximetry: Can necessity become the mother of intervention? *Annals of the American Thoracic Society* **2021**, *18*, 952-954, doi:https://dx.doi.org/10.1513/AnnalsATS.202103-281ED.
26. Fernandez-Fernandez, F.J. Electrocardiographic predictors of atrial fibrillation: prolonged P-wave and Bayes syndrome. *Journal of hypertension* **2019**, *37*, 454-455, doi:https://dx.doi.org/10.1097/HJH.0000000000001990.
27. Filos, D.; Chouvarda, I.; Tachmatzidis, D.; Vassilikos, V.; Maglaveras, N. Beat-to-beat P-wave morphology as a predictor of paroxysmal atrial fibrillation. *Computer methods and programs in biomedicine* **2017**, *151*, 111-121, doi:https://dx.doi.org/10.1016/j.cmpb.2017.08.016.
28. ElMoaqet, H.; Almuwaqat, Z.; Ryalat, M.; Almtireen, N. A New Algorithm for Short Term Prediction of Persistent Atrial Fibrillation. In Proceedings of the 2017 IEEE JORDAN CONFERENCE ON APPLIED ELECTRICAL ENGINEERING AND COMPUTING TECHNOLOGIES (AEECT), 2017.
29. Firyulina, M.A.; Kashirina, I.L.; Iop. Classification of cardiac arrhythmia using machine learning techniques. In Proceedings of the APPLIED MATHEMATICS, COMPUTATIONAL SCIENCE AND MECHANICS: CURRENT PROBLEMS, 2020.
30. Fujita, H.; Cimr, D. Decision support system for arrhythmia prediction using convolutional neural network structure without preprocessing. *APPLIED INTELLIGENCE* **2019**, *49*, 3383-3391, doi:10.1007/s10489-019-01461-0.
31. Gilon, C.; Gregoire, J.M.; Bersini, H.; Ieee. Forecast of paroxysmal atrial fibrillation using a deep neural network. In Proceedings of the 2020 INTERNATIONAL JOINT CONFERENCE ON NEURAL NETWORKS (IJCNN), 2020.
32. Gonzalez-Cordero, A.F.; Duconge-Soler, J.; Franqui-Rivera, H.; Feliu-Maldonado, R.; Roche-Lima, A.; Almodovar-Rivera, I. Insight on the Genetics of Atrial Fibrillation in Puerto Rican Hispanics. *Stroke research and treatment* **2021**, *2021*, 8819896, doi:https://dx.doi.org/10.1155/2021/8819896.
33. Gregoire, J.; Gilon, C.; Subramanian, N.; Bersini, H. Forecasting episodes of atrial fibrillation using a new machine learning algorithm. *Archives of Cardiovascular Diseases Supplements* **2020**, *12*, 103, doi:https://dx.doi.org/10.1016/j.acvdsp.2019.09.225.
34. Gregoire, J.-M.; Gilon, C.; Carlier, S.; Bersini, H. Role of the autonomic nervous system and premature atrial contractions in short-term paroxysmal atrial fibrillation forecasting: Insights from machine learning models. *Archives of cardiovascular diseases* **2022**, *115*, 377-387, doi:https://dx.doi.org/10.1016/j.acvd.2022.04.006.
35. Gregoire, J.M.; Gilon, C.; Carlier, S.; Bersini, H. Unravelling the black box of machine learning for atrial fibrillation forecast: Role of heart rate variability and of premature beats. *European Heart Journal* **2020**, *41*, 671, doi:https://dx.doi.org/10.1093/ehjci/ehaa946.0671.
36. Gregoire, J.M.; Gilon, C.; Carlier, S.; Bersini, H. Influence of autonomic nervous system on atrial fibrillation trigger, as assessed by machine learning. *Acta Cardiologica* **2021**, *76*, 12, doi:https://dx.doi.org/10.1080/00015385.2021.1880175.

37. Gregoire, J.M.; Subramanian, N.; Papazian, D.; Bersini, H. Forecasting atrial fibrillation using machine learning techniques. *European Heart Journal* **2019**, *40*, 4163, doi:https://dx.doi.org/10.1093/eurheartj/ehz746.1157.
38. Grout, R.W.; Hui, S.L.; Imler, T.D.; El-Azab, S.; Baker, J.; Sands, G.H.; Ateya, M.; Pike, F. Development, validation, and proof-of-concept implementation of a two-year risk prediction model for undiagnosed atrial fibrillation using common electronic health data (UNAFIED). *BMC medical informatics and decision making* **2021**, *21*, 112, doi:https://dx.doi.org/10.1186/s12911-021-01482-1.
39. Guo, Y.T.; Cui, Y.; Zhao, C.; Liu, L.; Li, L.; Chen, M. Machine-learning fusion approach for the prediction of atrial fibrillation onset using photoplethysmographic-based smart device. *European Heart Journal* **2021**, *42*, 3058, doi:https://dx.doi.org/10.1093/eurheartj/ehab724.3058.
40. Hayn, D.; Kollmann, A.; Schreier, G. Predicting initiation and termination of atrial fibrillation from the ECG. *Biomedizinische Technik. Biomedical engineering* **2007**, *52*, 5-10.
41. Henry, C.; Singh, J.P.; Fontanarava, J.; de Masse, G.; Carbonati, T.; Gardella, C.; Fiorina, L.; Li, J. SHORT TERM PREDICTION OF ATRIAL FIBRILLATION FROM AMBULATORY ECG USING DEEP LEARNING. *Journal of the American College of Cardiology* **2022**, *79*, 2015, doi:https://dx.doi.org/10.1016/S0735-1097%2822%2903006-6.
42. Heo, T.S.; Kim, C.; Kim, J.D.; Park, C.Y.; Kim, Y.S. Prediction of Atrial Fibrillation Cases: Convolutional Neural Networks Using the Output Texts of Electrocardiography. *SENSORS AND MATERIALS* **2021**, *33*, 393-404, doi:10.18494/SAM.2021.3023.
43. Hill, N.R.; Ayoubkhani, D.; Lumley, M.; Lister, S.; Farooqui, U.; Clifton, D.; O'Neil, M.; McEwan, P.; Gordon, J. Machine learning to detect and diagnose atrial fibrillation and atrial flutter (AF/F) using routine clinical data. *Value in Health* **2018**, *21*, S213.
44. Hill, N.R.; Ayoubkhani, D.; McEwan, P.; Sugrue, D.M.; Farooqui, U.; Lister, S.; Lumley, M.; Bakhai, A.; Cohen, A.T.; O'Neill, M.; et al. Predicting atrial fibrillation in primary care using machine learning. *PloS one* **2019**, *14*, e0224582, doi:https://dx.doi.org/10.1371/journal.pone.0224582.
45. Hill, N.R.; Arden, C.; Beresford-Hulme, L.; Camm, A.J.; Clifton, D.; Davies, D.W.; Farooqui, U.; Gordon, J.; Groves, L.; Hurst, M.; et al. Identification of undiagnosed atrial fibrillation patients using a machine learning risk prediction algorithm and diagnostic testing (PULsE-AI): Study protocol for a randomised controlled trial. *Contemporary clinical trials* **2020**, *99*, 106191, doi:https://dx.doi.org/10.1016/j.cct.2020.106191.
46. Hirota, N.; Suzuki, S.; Arita, T.; Yagi, N.; Otsuka, T.; Semba, H.; Kano, H.; Matsuno, S.; Kato, Y.; Uejima, T.; et al. Prediction of atrial fibrillation by 12-lead electrocardiogram parameters in patients without structural heart disease. *European Heart Journal* **2020**, *41*, 536, doi:https://dx.doi.org/10.1093/ehjci/ehaa946.0536.
47. Hong, S.D.; Wang, C.; Fu, Z.J. Gated temporal convolutional neural network and expert features for diagnosing and explaining physiological time series: A case study on heart rates. *COMPUTER METHODS AND PROGRAMS IN BIOMEDICINE* **2021**, *200*, doi:10.1016/j.cmpb.2020.105847.
48. Hsieh, J.-C.; Shih, H.; Xin, L.-L.; Yang, C.-C.; Han, C.-L. 12-lead ECG signal processing and atrial fibrillation prediction in clinical practice. *Technology and health*

care : official journal of the European Society for Engineering and Medicine **2022**, doi:https://dx.doi.org/10.3233/THC-212925.
49. Hu, W.S. TCTAP A-001 A Novel Atrial Fibrillation Prediction Model for Asian Subjects - A Nationwide Cohort Study. *Journal of the American College of Cardiology* **2019**, *73*, S1, doi:https://dx.doi.org/10.1016/j.jacc.2019.03.026.
50. Jalali, A.; Lee, M. Atrial Fibrillation Prediction With Residual Network Using Sensitivity and Orthogonality Constraints. *IEEE journal of biomedical and health informatics* **2020**, *24*, 407-413, doi:https://dx.doi.org/10.1109/JBHI.2019.2957809.
51. Jyothi, S.; Nelloru, G. Predicting arrhythmia, atrial fibrillation from electrocardiogram signals using Pivot Range Fitness Scale-Based Machine Learning Model. *INTERNATIONAL JOURNAL OF INTELLIGENT UNMANNED SYSTEMS*, doi:10.1108/IJIUS-11-2021-0140.
52. Karnik, S.; Tan, S.L.; Berg, B.; Glurich, I.; Zhang, J.F.; Vidaillet, H.J.; Page, C.D.; Chowdhary, E.; Ieee. Predicting Atrial Fibrillation and Flutter using Electronic Health Records. In Proceedings of the 2012 ANNUAL INTERNATIONAL CONFERENCE OF THE IEEE ENGINEERING IN MEDICINE AND BIOLOGY SOCIETY (EMBC), 2012; pp. 5562-5565.
53. Kasturiwale, H.P.; Kale, S.N. BioSignal modelling for prediction of cardiac diseases using intra group selection method. *INTELLIGENT DECISION TECHNOLOGIES-NETHERLANDS* **2021**, *15*, 151-160, doi:10.3233/IDT-200058.
54. Kawakami, M.; Karashima, S.; Morita, K.; Tada, H.; Okada, H.; Aono, D.; Kometani, M.; Nomura, A.; Demura, M.; Furukawa, K.; et al. Explainable Machine Learning for Atrial Fibrillation in the General Population Using a Generalized Additive Model - A Cross-Sectional Study. *Circulation reports* **2022**, *4*, 73-82, doi:https://dx.doi.org/10.1253/circrep.CR-21-0151.
55. Kim, I.S.; Yang, P.S.; Yu, H.T.; Kim, T.H.; Uhm, J.S.; Pak, H.N.; Lee, M.H.; Kim, J.Y.; Joung, B. Clinical applications of machine learning for prediction of incident atrial fibrillation from the general population: A nationwide cohort study. *European Heart Journal* **2019**, *40*, 3579, doi:https://dx.doi.org/10.1093/eurheartj/ehz746.0651.
56. Kim, I.-S.; Yang, P.-S.; Jang, E.; Jung, H.; You, S.C.; Yu, H.T.; Kim, T.-H.; Uhm, J.-S.; Pak, H.-N.; Lee, M.-H.; et al. Long-term PM2.5 exposure and the clinical application of machine learning for predicting incident atrial fibrillation. *Sci Rep* **2020**, *10*, 16324, doi:10.1038/s41598-020-73537-8.
57. Kim, M.; Kang, Y.; You, S.C.; Park, H.-D.; Lee, S.-S.; Kim, T.-H.; Yu, H.T.; Choi, E.-K.; Park, H.-S.; Park, J.; et al. Artificial intelligence predicts clinically relevant atrial high-rate episodes in patients with cardiac implantable electronic devices. *Scientific reports* **2022**, *12*, 37, doi:https://dx.doi.org/10.1038/s41598-021-03914-4.
58. Kordik, P.; Kremen, V.; Lhotska, L. The GAME algorithm applied to complex fractionated atrial electrograms data set. In Proceedings of the ARTIFICIAL NEURAL NETWORKS - ICANN 2008, PT II, 2008; pp. 859-+.
59. Krstacic, G.; Gamberger, D.; Smuc, T.; Krstacic, A.; Ieee; Ieee. Some important R-R interval based paroxysmal atrial fibrillation predictors. In Proceedings of the COMPUTERS IN CARDIOLOGY 2001, VOL 28, 2001; pp. 409-412.
60. Kwon, O.-S.; Hong, M.; Kim, T.-H.; Hwang, I.; Shim, J.; Choi, E.-K.; Lim, H.E.; Yu, H.T.; Uhm, J.-S.; Joung, B.; et al. Genome-wide association study-based prediction of atrial fibrillation using artificial intelligence. *Open heart* **2022**, *9*, doi:https://dx.doi.org/10.1136/openhrt-2021-001898.

61. Lehtonen, A.O.; Niiranen, T.J. Electrocardiographic predictors of atrial fibrillation: prolonged P-wave and Bayes syndrome Reply. *JOURNAL OF HYPERTENSION* **2019**, *37*, 455-455, doi:10.1097/HJH.0000000000001991.
62. Li, Z.; Derksen, H.; Gryak, J.; Jiang, C.; Gao, Z.; Zhang, W.; Ghanbari, H.; Gunaratne, P.; Najarian, K. Prediction of cardiac arrhythmia using deterministic probabilistic finite-state automata. *Biomedical Signal Processing and Control* **2021**, *63*, 102200, doi:https://dx.doi.org/10.1016/j.bspc.2020.102200.
63. Liu, X.L.; Liu, T.B.; Zhang, Z.B.; Kuo, P.C.; Xu, H.R.; Yang, Z.C.; Lan, K.; Li, P.Y.; Ouyang, Z.C.; Ng, Y.L.; et al. TOP-Net Prediction Model Using Bidirectional Long Short-term Memory and Medical-Grade Wearable Multisensor System for Tachycardia Onset: Algorithm Development Study. *JMIR MEDICAL INFORMATICS* **2021**, *9*, doi:10.2196/18803.
64. Liu, Y.; Liu, N.; Bai, F.; Liu, Q. Identifying ceRNA Networks Associated With the Susceptibility and Persistence of Atrial Fibrillation Through Weighted Gene Co-Expression Network Analysis. *Frontiers in genetics* **2021**, *12*, 653474, doi:https://dx.doi.org/10.3389/fgene.2021.653474.
65. Loring, Z.; Mehrotra, S.; Piccini, J.P.; Camm, J.; Carlson, D.; Fonarow, G.C.; Fox, K.A.A.; Peterson, E.D.; Pieper, K.; Kakkar, A.K. Machine learning does not improve upon traditional regression in predicting outcomes in atrial fibrillation: an analysis of the ORBIT-AF and GARFIELD-AF registries. *Europace : European pacing, arrhythmias, and cardiac electrophysiology : journal of the working groups on cardiac pacing, arrhythmias, and cardiac cellular electrophysiology of the European Society of Cardiology* **2020**, *22*, 1635-1644, doi:https://dx.doi.org/10.1093/europace/euaa172.
66. Lou, Y.-S.; Lin, C.-S.; Fang, W.-H.; Lee, C.-C.; Ho, C.-L.; Wang, C.-H.; Lin, C. Artificial Intelligence-Enabled Electrocardiogram Estimates Left Atrium Enlargement as a Predictor of Future Cardiovascular Disease. *Journal of personalized medicine* **2022**, *12*, doi:https://dx.doi.org/10.3390/jpm12020315.
67. Maghawry, E.; Ismail, R.; Gharib, T.E. An efficient approach for Paroxysmal Atrial Fibrillation events prediction using Extreme Learning Machine. *JOURNAL OF INTELLIGENT & FUZZY SYSTEMS* **2021**, *40*, 5087-5099, doi:10.3233/JIFS-201832.
68. McMillan, S.; Bauman, Z.; Horst, H.M.; Gassner, M.; Blyden, D.; Syed, Z.; Rubinfeld, I. Computational prediction of atrial fibrillation in the intensive care unit using early admission vital signs data. *Critical Care Medicine* **2012**, *40*, 60, doi:https://dx.doi.org/10.1097/01.ccm.0000425605.04623.4b.
69. Melzi, P.; Tolosana, R.; Cecconi, A.; Sanz-Garcia, A.; Ortega, G.J.; Jimenez-Borreguero, L.J.; Vera-Rodriguez, R. Analyzing artificial intelligence systems for the prediction of atrial fibrillation from sinus-rhythm ECGs including demographics and feature visualization. *Scientific reports* **2021**, *11*, 22786, doi:https://dx.doi.org/10.1038/s41598-021-02179-1.
70. Mendez, M.M.; Hsu, M.C.; Yuan, J.T.; Lynn, K.S. A Heart Rate Variability-Based Paroxysmal Atrial Fibrillation Prediction System. *APPLIED SCIENCES-BASEL* **2022**, *12*, doi:10.3390/app12052387.
71. Mohebbi, M.; Ghassemian, H. Prediction of paroxysmal atrial fibrillation using recurrence plot-based features of the RR-interval signal. *Physiological measurement* **2011**, *32*, 1147-1162, doi:https://dx.doi.org/10.1088/0967-3334/32/8/010.
72. Mohebbi, M.; Ghassemian, H. Prediction of paroxysmal atrial fibrillation based on non-linear analysis and spectrum and bispectrum features of the heart rate

variability signal. *Computer methods and programs in biomedicine* **2012**, *105*, 40-49, doi:https://dx.doi.org/10.1016/j.cmpb.2010.07.011.
73. Mohebbi, M.; Ghassemian, H.; Asl, B.M. Structures of the recurrence plot of heart rate variability signal as a tool for predicting the onset of paroxysmal atrial fibrillation. *Journal of medical signals and sensors* **2011**, *1*, 113-121.
74. Mroueh, M.; Mourad-Chehade, F.; Abdallah, F. Evidence-based prediction of Atrial Fibrillation using physiological signals. In Proceedings of the 2019 3RD INTERNATIONAL CONFERENCE ON BIO-ENGINEERING FOR SMART TECHNOLOGIES (BIOSMART), 2019.
75. Nadarajah, R.; Wu, J.; Frangi, A.F.; Hogg, D.; Cowan, C.; Gale, C. Predicting patient-level new-onset atrial fibrillation from population-based nationwide electronic health records: protocol of FIND-AF for developing a precision medicine prediction model using artificial intelligence. *BMJ open* **2021**, *11*, e052887, doi:https://dx.doi.org/10.1136/bmjopen-2021-052887.
76. Okutucu, S.; Katircioglu-Ozturk, D.; Oto, E.; Guvenir, H.A.; Karaagaoglu, E.; Oto, A.; Meinertz, T.; Goette, A. Data mining experiments on the Angiotensin II-Antagonist in Paroxysmal Atrial Fibrillation (ANTIPAF-AFNET 2) trial: 'exposing the invisible'. *Europace : European pacing, arrhythmias, and cardiac electrophysiology : journal of the working groups on cardiac pacing, arrhythmias, and cardiac cellular electrophysiology of the European Society of Cardiology* **2017**, *19*, 741-746, doi:https://dx.doi.org/10.1093/europace/euw084.
77. Parsi, A.; Glavin, M.; Jones, E.; Byrne, D. Prediction of paroxysmal atrial fibrillation using new heart rate variability features. *Computers in biology and medicine* **2021**, *133*, 104367, doi:https://dx.doi.org/10.1016/j.compbiomed.2021.104367.
78. Ponomartseva, D.A.; Derevitskii, I.V.; Kovalchuk, S.V.; Babenko, A.Y. Prediction model for thyrotoxic atrial fibrillation: a retrospective study. *BMC endocrine disorders* **2021**, *21*, 150, doi:https://dx.doi.org/10.1186/s12902-021-00809-3.
79. Pourbabaee, B.; Lucas, C.; Ieee. AUTOMATIC DETECTION AND PREDICTION OF PAROXYSMAL ATRIAL FIBRILLATION BASED ON ANALYZING ECG SIGNAL FEATURE CLASSIFICATION METHODS. In Proceedings of the 2008 CAIRO INTERNATIONAL BIOMEDICAL ENGINEERING CONFERENCE, 2008; pp. 193-196.
80. Raghunath, S.M.; Cerna, A.U.; Jing, L.; VanMaanen, D.; Stough, J.V.; Hartzel, D.; Leader, J.; Good, C.; Fornwalt, B.K.; Haggerty, C.M. A deep neural network for predicting incident atrial fibrillation directly from 12-lead electrocardiogram traces. *Circulation* **2019**, *140*, doi:https://dx.doi.org/10.1161/circ.140.suppl_1.14407.
81. Raghunath, S.; Pfeifer, J.M.; Ulloa-Cerna, A.E.; Nemani, A.; Carbonati, T.; Jing, L.; vanMaanen, D.P.; Hartzel, D.N.; Ruhl, J.A.; Lagerman, B.F.; et al. Deep Neural Networks Can Predict New-Onset Atrial Fibrillation From the 12-Lead ECG and Help Identify Those at Risk of Atrial Fibrillation-Related Stroke. *Circulation* **2021**, *143*, 1287-1298, doi:https://dx.doi.org/10.1161/CIRCULATIONAHA.120.047829.
82. Ramirez, J.; Duijvenboden, S.V.; Young, W.J.; Orini, M.; Jones, A.R.; Lambiase, P.D.; Munroe, P.B.; Tinker, A. Analysing electrocardiographic traits and predicting cardiac risk in UK biobank. *JRSM Cardiovascular Disease* **2021**, *10*, doi:https://dx.doi.org/10.1177/20480040211023664.
83. Ravens, U.; Katircioglu-Ozturk, D.; Wettwer, E.; Christ, T.; Dobrev, D.; Voigt, N.; Poulet, C.; Loose, S.; Simon, J.; Stein, A.; et al. Application of the RIMARC algorithm

to a large data set of action potentials and clinical parameters for risk prediction of atrial fibrillation. *Medical & biological engineering & computing* **2015**, *53*, 263-273, doi:https://dx.doi.org/10.1007/s11517-014-1232-0.
84. Ravish, D.K.; Shenoy, N.R.; Shanthi, K.J.; Nisargh, S.; Ieee. Heart Function Monitoring, Prediction and Prevention of Heart Attacks: Using Artificial Neural Networks. In Proceedings of the 2014 INTERNATIONAL CONFERENCE ON CONTEMPORARY COMPUTING AND INFORMATICS (IC3I), 2014; pp. 1-6.
85. Safabakhsh, S.; Zhao, R.; Laksman, Z. APPLYING A DEEP NEURAL NETWORK FOR AUTOMATED PREDICTION OF PAROXYSMAL ATRIAL FIBRILLATION ONSET. *Canadian Journal of Cardiology* **2020**, *36*, S42-S43, doi:https://dx.doi.org/10.1016/j.cjca.2020.07.094.
86. Shen, M.T.; Zhang, L.Q.; Luo, X.; Xu, J.; Publishing, I.O.P. Atrial Fibrillation Prediction Algorithm Based on Attention Model. In Proceedings of the 5TH ANNUAL INTERNATIONAL CONFERENCE ON INFORMATION SYSTEM AND ARTIFICIAL INTELLIGENCE (ISAI2020), 2020.
87. Sovilj, S.; Rajsman, G.; Magjarevic, R. ECG based prediction of Atrial Fibrillation using Support Vector Classifier. *AUTOMATIKA* **2011**, *52*, 58-67.
88. Sun, L.; Wang, Y.; He, J.; Li, H.; Peng, D.; Wang, Y. A stacked LSTM for atrial fibrillation prediction based on multivariate ECGs. *Health information science and systems* **2020**, *8*, 19, doi:https://dx.doi.org/10.1007/s13755-020-00103-x.
89. Surucu, M.; Isler, Y.; Perc, M.; Kara, R. Convolutional neural networks predict the onset of paroxysmal atrial fibrillation: Theory and applications. *Chaos (Woodbury, N.Y.)* **2021**, *31*, 113119, doi:https://dx.doi.org/10.1063/5.0069272.
90. Suzuki, S.; Motogi, J.; Nakai, H.; Matsuzawa, W.; Takayanagi, T.; Umemoto, T.; Hirota, N.; Hyodo, A.; Satoh, K.; Otsuka, T.; et al. Identifying patients with atrial fibrillation during sinus rhythm on ECG: Significance of the labeling in the artificial intelligence algorithm. *International journal of cardiology. Heart & vasculature* **2022**, *38*, 100954, doi:https://dx.doi.org/10.1016/j.ijcha.2022.100954.
91. Szep, J.; Hariri, S.; Khalpey, Z.; Ieee. PREDICTIVE DIAGNOSIS OF FATAL HEART RHYTHMS USING WEARABLES. In Proceedings of the 2019 SPRING SIMULATION CONFERENCE (SPRINGSIM), 2019.
92. Tabassum, T.; Islam, M.; Ieee. An Approach of Cardiac Disease Prediction by Analyzing ECG Signal. In Proceedings of the 2016 3RD INTERNATIONAL CONFERENCE ON ELECTRICAL ENGINEERING AND INFORMATION & COMMUNICATION TECHNOLOGY (ICEEICT), 2016.
93. Taggar, J.S.; Qureshi, N.; Weng, S. Risk prediction of new AF: is there a role for artificial intelligence? *European journal of preventive cardiology* **2020**, *27*, 1325-1327, doi:https://dx.doi.org/10.1177/2047487319879525.
94. Talukdar, J.; Dewangan, B.K. Analysis of cardiovascular diseases using artificial neural network. In Proceedings of the 2018 FIFTH INTERNATIONAL CONFERENCE ON PARALLEL, DISTRIBUTED AND GRID COMPUTING (IEEE PDGC), 2018; pp. 132-137.
95. Tieleman, R.G. [Predicting atrial fibrillation through a sinus-rhythm electrocardiogram; useful or not?]. *Atriumfibrilleren voorspellen met een sinusritme-ecg; wel of niet zinning?* **2019**, *163*.
96. Tse, G.; Lakhani, I.; Zhou, J.; Li, K.H.C.; Lee, S.; Liu, Y.; Leung, K.S.K.; Liu, T.; Baranchuk, A.; Zhang, Q. P-Wave Area Predicts New Onset Atrial Fibrillation in

Mitral Stenosis: A Machine Learning Approach. *Frontiers in bioengineering and biotechnology* **2020**, *8*, 479, doi:https://dx.doi.org/10.3389/fbioe.2020.00479.
97. Tzou, H.-A.; Lin, S.-F.; Chen, P.-S. Paroxysmal atrial fibrillation prediction based on morphological variant P-wave analysis with wideband ECG and deep learning. *Computer methods and programs in biomedicine* **2021**, *211*, 106396, doi:https://dx.doi.org/10.1016/j.cmpb.2021.106396.
98. Wang, L.-H.; Yan, Z.-H.; Yang, Y.-T.; Chen, J.-Y.; Yang, T.; Kuo, I.C.; Abu, P.A.R.; Huang, P.-C.; Chen, C.-A.; Chen, S.-L. A Classification and Prediction Hybrid Model Construction with the IQPSO-SVM Algorithm for Atrial Fibrillation Arrhythmia. *Sensors (Basel, Switzerland)* **2021**, *21*, doi:https://dx.doi.org/10.3390/s21155222.
99. Wang, X.; Khurshid, S.; Choi, S.H.; Friedman, S.; Weng, L.C.; Reeder, C.; Pirruccello, J.P.; Singh, P.; Lau, E.S.; Venn, R.; et al. Genetic Susceptibility to Atrial Fibrillation Identified via Deep Learning of 12-lead Electrocardiograms. *medRxiv* **2022**, doi:https://dx.doi.org/10.1101/2022.01.17.22269357.
100. Wang, X.; Meng, X.; Meng, L.; Guo, Y.; Li, Y.; Yang, C.; Pei, Z.; Li, J.; Wang, F. Joint efficacy of the three biomarkers SNCA, GYPB and HBG1 for atrial fibrillation and stroke: Analysis via the support vector machine neural network. *Journal of Cellular and Molecular Medicine* **2022**, *26*, 2010-2022, doi:https://dx.doi.org/10.1111/jcmm.17224.
101. Wehbe, R.M.; Khan, S.S.; Shah, S.J.; Ahmad, F.S. Predicting High-Risk Patients and High-Risk Outcomes in Heart Failure. *HEART FAILURE CLINICS* **2020**, *16*, 387-407, doi:10.1016/j.hfc.2020.05.002.
102. Wu, C.; Hwang, M.; Huang, T.-H.; Chen, Y.-M.J.; Chang, Y.-J.; Ho, T.-H.; Huang, J.; Hwang, K.-S.; Ho, W.-H. Application of artificial intelligence ensemble learning model in early prediction of atrial fibrillation. *BMC bioinformatics* **2021**, *22*, 93, doi:https://dx.doi.org/10.1186/s12859-021-04000-2.
103. Xie, E.; Wu, C.; Ostovaneh, M.; Post, W.S.; Kutty, S.; Soliman, E.Z.; Bluemke, D.A.; Heckbert, S.R.; Lima, J.; Ambale-Venkatesh, B. Intermediate Markers Underlying Electrocardiographic Predictors of Incident Atrial Fibrillation: The MESA. *Circulation. Arrhythmia and electrophysiology* **2021**, *14*, e009805, doi:https://dx.doi.org/10.1161/CIRCEP.121.009805.
104. Xin, Y.; Zhao, Y. Paroxysmal atrial fibrillation recognition based on multi-scale wavelet alpha-entropy. *Biomedical engineering online* **2017**, *16*, 121, doi:https://dx.doi.org/10.1186/s12938-017-0406-z.
105. Yang, L.; Chen, Y.; Huang, W. Hub Genes Identification, Small Molecule Compounds Prediction for Atrial Fibrillation and Diagnostic Model Construction Based on XGBoost Algorithm. *Frontiers in cardiovascular medicine* **2022**, *9*, 920399, doi:https://dx.doi.org/10.3389/fcvm.2022.920399.
106. Ye, X.H.; Huang, Y.Q.; Lu, Q. Explainable Prediction of Cardiac Arrhythmia Using Machine Learning. In Proceedings of the 2021 14TH INTERNATIONAL CONGRESS ON IMAGE AND SIGNAL PROCESSING, BIOMEDICAL ENGINEERING AND INFORMATICS (CISP-BMEI 2021), 2021.
107. Zhang, H.; Dong, Z.; Gao, J.; Lu, P.; Wang, Z. Automatic screening method for atrial fibrillation based on lossy compression of the electrocardiogram signal. *Physiological measurement* **2020**, *41*, 075005, doi:https://dx.doi.org/10.1088/1361-6579/ab979f.
108. Zhang, P.; Miao, Q.; Wang, X.; Zhang, Y.; Hou, Y. Identification of a Novel 4-gene Diagnostic Model for Atrial Fibrillation Risk Based on Integrated Analysis Across

Independent Data Sets. *Combinatorial chemistry & high throughput screening* **2022**, *25*, 229-240, doi:https://dx.doi.org/10.2174/1386207324666210121103304.
109. Ahmad, A.; Mansour, S.; Zgheib, A.; Safatly, L.; Hajj, A.E.; Baydoun, M.; Ghaziri, H.; Aridi, H.; Ismaeel, H. USING ARTIFICIAL INTELLIGENCE TO UNCOVER ASSOCIATION OF LEFT ATRIAL STRAIN WITH THE FRAMINGHAM RISK SCORE FOR ATRIAL FIBRILLATION DEVELOPMENT. *Journal of the American College of Cardiology* **2020**, *75*, 455, doi:https://dx.doi.org/10.1016/S0735-1097%2820%2931082-2.
110. Ambale-Venkatesh, B.; Yang, X.; Wu, C.O.; Liu, K.; Hundley, W.G.; McClelland, R.; Gomes, A.S.; Folsom, A.R.; Shea, S.; Guallar, E.; et al. Cardiovascular Event Prediction by Machine Learning: The Multi-Ethnic Study of Atherosclerosis. *Circulation research* **2017**, *121*, 1092-1101, doi:https://dx.doi.org/10.1161/CIRCRESAHA.117.311312.
111. Christopoulos, G.; Graff-Radford, J.; Lopez, C.L.; Yao, X.; Attia, Z.I.; Rabinstein, A.A.; Petersen, R.C.; Knopman, D.S.; Mielke, M.M.; Kremers, W.; et al. Artificial Intelligence-Electrocardiography to Predict Incident Atrial Fibrillation: A Population-Based Study. *Circulation. Arrhythmia and electrophysiology* **2020**, *13*, e009355, doi:https://dx.doi.org/10.1161/CIRCEP.120.009355.
112. Hirota, N.; Suzuki, S.; Arita, T.; Yagi, N.; Otsuka, T.; Kishi, M.; Semba, H.; Kano, H.; Matsuno, S.; Kato, Y.; et al. Prediction of current and new development of atrial fibrillation on electrocardiogram with sinus rhythm in patients without structural heart disease. *International journal of cardiology* **2021**, *327*, 93-99, doi:https://dx.doi.org/10.1016/j.ijcard.2020.11.012.
113. Hu, W.S.; Hsieh, M.H.; Lin, C.L. A novel atrial fibrillation prediction model for Chinese subjects: A nationwide cohort investigation of 682 237 study participants with random forest model. *Europace* **2019**, *21*, 1307-1312, doi:https://dx.doi.org/10.1093/europace/euz036.
114. Joo, G.; Song, Y.; Im, H.; Park, J. Clinical Implication of Machine Learning in Predicting the Occurrence of Cardiovascular Disease Using Big Data (Nationwide Cohort Data in Korea). *IEEE ACCESS* **2020**, *8*, 157643-157653, doi:10.1109/ACCESS.2020.3015757.
115. Kaminski, A.E.; Albus, M.L.; Ball, C.T.; White, L.J.; Sheele, J.M.; Attia, Z.I.; Friedman, P.A.; Adedinsewo, D.A.; Noseworthy, P.A. Evaluating atrial fibrillation artificial intelligence for the ED: statistical and clinical implications. *The American journal of emergency medicine* **2022**, *57*, 98-102, doi:https://dx.doi.org/10.1016/j.ajem.2022.04.032.
116. Khurshid, S.; Friedman, S.; Reeder, C.; Di Achille, P.; Diamant, N.; Singh, P.; Harrington, L.X.; Wang, X.; Al-Alusi, M.A.; Sarma, G.; et al. ECG-Based Deep Learning and Clinical Risk Factors to Predict Atrial Fibrillation. *Circulation* **2022**, *145*, 122-133, doi:https://dx.doi.org/10.1161/CIRCULATIONAHA.121.057480.
117. Kim, K.; Park, S.M. Artificial neural networks to compare the contribution of basic clinical factors, ESC SCORE, and multidimensional risk factors for cardiovascular event prediction performance: An observational study. *European Heart Journal* **2020**, *41*, 2897, doi:https://dx.doi.org/10.1093/ehjci/ehaa946.2897.
118. Lip, G.Y.H.; Genaidy, A.; Tran, G.; Marroquin, P.; Estes, C. Incidence and Complications of Atrial Fibrillation in a Low Socioeconomic and High Disability United States (US) Population: A Combined Statistical and Machine Learning

Approach. *International journal of clinical practice* **2022**, *2022*, 8649050, doi:https://dx.doi.org/10.1155/2022/8649050.
119. Rabinstein, A.A.; Yost, M.D.; Faust, L.; Kashou, A.H.; Latif, O.S.; Graff-Radford, J.; Attia, I.Z.; Yao, X.; Noseworthy, P.A.; Friedman, P.A. Artificial Intelligence-Enabled ECG to Identify Silent Atrial Fibrillation in Embolic Stroke of Unknown Source. *Journal of stroke and cerebrovascular diseases : the official journal of National Stroke Association* **2021**, *30*, 105998, doi:https://dx.doi.org/10.1016/j.jstrokecerebrovasdis.2021.105998.
120. Reinke, F.; Bettin, M.; Ross, L.S.; Kochhäuser, S.; Kleffner, I.; Ritter, M.; Minnerup, J.; Dechering, D.; Eckardt, L.; Dittrich, R. Refinement of detecting atrial fibrillation in stroke patients: results from the TRACK-AF Study. *European Journal of Neurology* **2018**, *25*, 631-636, doi:https://doi.org/10.1111/ene.13538.
121. Schnabel, R.B.; Witt, H.; Walker, J.; Ludwig, M.; Geelhoed, B.; Kossack, N.; Schild, M.; Miller, R.; Kirchhof, P. Machine learning-based identification of risk-factor signatures for undiagnosed atrial fibrillation in primary prevention and post-stroke in clinical practice. *European heart journal. Quality of care & clinical outcomes* **2022**, doi:https://dx.doi.org/10.1093/ehjqcco/qcac013.
122. Sekelj, S.; Sandler, B.; Johnston, E.; Pollock, K.G.; Hill, N.R.; Gordon, J.; Tsang, C.; Khan, S.; Ng, F.S.; Farooqui, U. Detecting undiagnosed atrial fibrillation in UK primary care: Validation of a machine learning prediction algorithm in a retrospective cohort study. *EUROPEAN JOURNAL OF PREVENTIVE CARDIOLOGY* **2021**, *28*, 598-605, doi:10.1177/2047487320942338.
123. Shan, S.M.; Tang, S.C.; Huang, P.W.; Lin, Y.M.; Huang, W.H.; Lai, D.M.; Wu, A.Y.; Ieee. Reliable PPG-based Algorithm in Atrial Fibrillation Detection. In Proceedings of the PROCEEDINGS OF 2016 IEEE BIOMEDICAL CIRCUITS AND SYSTEMS CONFERENCE (BIOCAS), 2016; pp. 340-343.
124. Tiwari, P.; Colborn, K.L.; Smith, D.E.; Xing, F.; Ghosh, D.; Rosenberg, M.A. Assessment of a Machine Learning Model Applied to Harmonized Electronic Health Record Data for the Prediction of Incident Atrial Fibrillation. *JAMA network open* **2020**, *3*, e1919396, doi:https://dx.doi.org/10.1001/jamanetworkopen.2019.19396.
125. Abdul-Kadir, N.A.; Mat Safri, N.; Othman, M.A. Dynamic ECG features for atrial fibrillation recognition. *Computer Methods and Programs in Biomedicine* **2016**, *136*, 143-150, doi:https://dx.doi.org/10.1016/j.cmpb.2016.08.021.
126. Bahrami Rad, A.; Galloway, C.; Treiman, D.; Xue, J.; Li, Q.; Sameni, R.; Albert, D.; Clifford, G.D. Atrial fibrillation detection in outpatient electrocardiogram monitoring: An algorithmic crowdsourcing approach. *PloS one* **2021**, *16*, e0259916, doi:https://dx.doi.org/10.1371/journal.pone.0259916.
127. Ben Itzhak, S.; Ricon, S.S.; Biton, S.; Behar, J.A.; Sobel, J.A. Effect of temporal resolution on the detection of cardiac arrhythmias using HRV features and machine learning. *PHYSIOLOGICAL MEASUREMENT* **2022**, *43*, doi:10.1088/1361-6579/ac6561.
128. Chanthercrob, J.; Mahattanatawee, S.; Teeramongkonrasmee, A.; Somboon, P.; Ieee. Development of Rhythm-based and Morphology-based Algorithm for Atrial Fibrillation Detection From Single Lead ECG Signal. In Proceedings of the 2020 8TH INTERNATIONAL ELECTRICAL ENGINEERING CONGRESS (IEECON), 2020.
129. Couceiro, R.; Henriques, J.; Paiva, R.P.; Antunes, M.; Carvalho, P. Physiologically motivated detection of Atrial Fibrillation. *Annual International Conference of the IEEE Engineering in Medicine and Biology Society. IEEE Engineering in Medicine and Biology*

Society. Annual International Conference **2017**, *2017*, 1278-1281, doi:https://dx.doi.org/10.1109/EMBC.2017.8037065.
130. Duverney, D.; Gaspoz, J.M.; Pichot, V.; Roche, F.; Brion, R.; Antoniadis, A.; Barthelemy, J.C. High accuracy of automatic detection of atrial fibrillation using wavelet transform of heart rate intervals. *PACE-PACING AND CLINICAL ELECTROPHYSIOLOGY* **2002**, *25*, 457-462, doi:10.1046/j.1460-9592.2002.00457.x.
131. Eerikainen, L.M.; Bonomi, A.G.; Schipper, F.; Dekker, L.R.C.; de Morree, H.M.; Vullings, R.; Aarts, R.M. Detecting Atrial Fibrillation and Atrial Flutter in Daily Life Using Photoplethysmography Data. *IEEE JOURNAL OF BIOMEDICAL AND HEALTH INFORMATICS* **2020**, *24*, 1610-1618, doi:10.1109/JBHI.2019.2950574.
132. Fan, X.; Yao, Q.; Cai, Y.; Miao, F.; Sun, F.; Li, Y. Multiscaled Fusion of Deep Convolutional Neural Networks for Screening Atrial Fibrillation from Single Lead Short ECG Recordings. *IEEE Journal of Biomedical and Health Informatics* **2018**, *22*, 1744-1753, doi:https://dx.doi.org/10.1109/JBHI.2018.2858789.
133. Faust, O.; Acharya, U.R. Automated classification of five arrhythmias and normal sinus rhythm based on RR interval signals. *EXPERT SYSTEMS WITH APPLICATIONS* **2021**, *181*, doi:10.1016/j.eswa.2021.115031.
134. Helfenbein, E.; Gregg, R.; Lindauer, J.; Zhou, S. An Automated Algorithm for the Detection of Atrial Fibrillation in the Presence of Paced Rhythms. In Proceedings of the COMPUTING IN CARDIOLOGY 2010, VOL 37, 2010; pp. 113-116.
135. Ivanovic, M.D.; Atanasoski, V.; Shvilkin, A.; Hadzievski, L.; Maluckov, A. Deep Learning Approach for Highly Specific Atrial Fibrillation and Flutter Detection based on RR Intervals. *Annual International Conference of the IEEE Engineering in Medicine and Biology Society. IEEE Engineering in Medicine and Biology Society. Annual International Conference* **2019**, *2019*, 1780-1783, doi:https://dx.doi.org/10.1109/EMBC.2019.8856806.
136. Jia, Z.G.; Shi, Y.Y.; Saba, S.; Hu, J.T. On-device Prior Knowledge Incorporated Learning for Personalized Atrial Fibrillation Detection. *ACM TRANSACTIONS ON EMBEDDED COMPUTING SYSTEMS* **2021**, *20*, doi:10.1145/3476987.
137. Kennedy, A.; Finlay, D.D.; Guldenring, D.; Bond, R.R.; Moran, K.; McLaughlin, J. Automated detection of atrial fibrillation using R-R intervals and multivariate-based classification. *JOURNAL OF ELECTROCARDIOLOGY* **2016**, *49*, 871-876, doi:10.1016/j.jelectrocard.2016.07.033.
138. Kim, J.Y.; Kim, K.G.; Tae, Y.; Chang, M.; Park, S.-J.; Park, K.-M.; On, Y.K.; Kim, J.S.; Lee, Y.; Jang, S.-W. An Artificial Intelligence Algorithm With 24 h Holter Monitoring for the Identification of Occult Atrial Fibrillation During Sinus Rhythm. *Frontiers in cardiovascular medicine* **2022**, *9*, 906780, doi:https://dx.doi.org/10.3389/fcvm.2022.906780.
139. Krol-Jozaga, B. Atrial fibrillation detection using convolutional neural networks on 2-dimensional representation of ECG signal. *BIOMEDICAL SIGNAL PROCESSING AND CONTROL* **2022**, *74*, doi:10.1016/j.bspc.2021.103470.
140. Lee, H.-C.; Chen, C.-Y.; Lee, S.-J.; Lee, M.-C.; Tsai, C.-Y.; Chen, S.-T.; Li, Y.-J. Exploiting exercise electrocardiography to improve early diagnosis of atrial fibrillation with deep learning neural networks. *Computers in biology and medicine* **2022**, *146*, 105584, doi:https://dx.doi.org/10.1016/j.compbiomed.2022.105584.
141. Lee, K.; Choi, H.O.; Min, S.D.; Lee, J.; Guptha, B.B.; Nam, Y. A Comparative Evaluation of Atrial Fibrillation Detection Methods in Koreans Based on Optical

Recordings Using a Smartphone. *IEEE ACCESS* **2017**, *5*, 11437-11443, doi:10.1109/ACCESS.2017.2700488.
142. Lee, K.; Kim, S.; Choi, H.O.; Lee, J.; Nam, Y. Analyzing electrocardiogram signals obtained from a nymi band to detect atrial fibrillation. *MULTIMEDIA TOOLS AND APPLICATIONS* **2020**, *79*, 15985-15999, doi:10.1007/s11042-018-7075-1.
143. Lei, N.; Kareem, M.; Moon, S.K.; Ciaccio, E.J.; Acharya, U.R.; Faust, O. Hybrid decision support to monitor atrial fibrillation for stroke prevention. *International Journal of Environmental Research and Public Health* **2021**, *18*, 1-19, doi:https://dx.doi.org/10.3390/ijerph18020813.
144. Leutheuser, H.; Gradl, S.; Eskofier, B.M.; Tobola, A.; Lang, N.; Anneken, L.; Arnold, M.; Achenbach, S.; Ieee. Arrhythmia Classification Using RR Intervals: Improvement With Sinusoidal Regression Feature. In Proceedings of the 2015 IEEE 12TH INTERNATIONAL CONFERENCE ON WEARABLE AND IMPLANTABLE BODY SENSOR NETWORKS (BSN), 2015.
145. Liaqat, S.; Dashtipour, K.; Zahid, A.; Assaleh, K.; Arshad, K.; Ramzan, N. Detection of Atrial Fibrillation Using a Machine Learning Approach. *INFORMATION* **2020**, *11*, doi:10.3390/info11120549.
146. Liu, X.; Yang, X.; Wang, D.; Wong, A.; Ma, L.; Li, L. VidAF: A Motion-Robust Model for Atrial Fibrillation Screening From Facial Videos. *IEEE Journal of Biomedical and Health Informatics* **2022**, *26*, 1672-1683, doi:https://dx.doi.org/10.1109/JBHI.2021.3124967.
147. Medic, G.; Kotsopoulos, N.; Connolly, M.P.; Lavelle, J.; Norlock, V.; Wadhwa, M.; Mohr, B.A.; Derkac, W.M. Mobile Cardiac Outpatient Telemetry Patch vs. Implantable Loop Recorder in Cryptogenic Stroke Patients in the US - Cost-Minimization Model. *Medical devices (Auckland, N.Z.)* **2021**, *14*, 445-458, doi:https://dx.doi.org/10.2147/MDER.S337142.
148. Mittal, S.; Oliveros, S.; Li, J.; Barroyer, T.; Henry, C.; Gardella, C. AI Filter Improves Positive Predictive Value of Atrial Fibrillation Detection by an Implantable Loop Recorder. *JACC: Clinical Electrophysiology* **2021**, *7*, 965-975, doi:https://dx.doi.org/10.1016/j.jacep.2020.12.006.
149. Mousavi, S.; Afghah, F.; Acharya, U.R. HAN-ECG: An interpretable atrial fibrillation detection model using hierarchical attention networks. *COMPUTERS IN BIOLOGY AND MEDICINE* **2020**, *127*, doi:10.1016/j.compbiomed.2020.104057.
150. Nguyen, Q.H.; Do, T.T.T.; Thoppan, A.; Chong, C.F.; Arya, I.; Maddi, K.M.; Pandey, S.; Balakrishnan, V.K.; Pham, H.N.; Nguyen, B.P.; et al. Effective Arrhythmia Detection using Majority Voting. In Proceedings of the PROCEEDINGS OF 2019 INTERNATIONAL CONFERENCE ON SYSTEM SCIENCE AND ENGINEERING (ICSSE), 2019; pp. 109-114.
151. Nuryani, N.; Harjito, B.; Yahya, I.; Lestari, A.; Ieee. Atrial Fibrillation Detection Using Support Vector Machine. In Proceedings of the PROCEEDING JOINT INTERNATIONAL CONFERENCE ON ELECTRIC VEHICULAR TECHNOLOGY AND INDUSTRIAL, MECHANICAL, ELECTRICAL, AND CHEMICAL ENGINEERING (ICEVT & IMECE), 2015; pp. 215-218.
152. Park, J.; Lee, S.; Jeon, M. Atrial fibrillation detection by heart rate variability in Poincare plot. *BIOMEDICAL ENGINEERING ONLINE* **2009**, *8*, doi:10.1186/1475-925X-8-38.

153. Pereira, T.; Ding, C.; Gadhoumi, K.; Tran, N.; Colorado, R.A.; Meisel, K.; Hu, X. Deep learning approaches for plethysmography signal quality assessment in the presence of atrial fibrillation. *PHYSIOLOGICAL MEASUREMENT* **2019**, *40*, doi:10.1088/1361-6579/ab5b84.
154. Pham, T.H.; Sree, V.; Mapes, J.; Dua, S.; Lih, O.S.; Koh, J.E.W.; Ciaccio, E.J.; Acharya, U.R. A novel machine learning framework for automated detection of arrhythmias in ECG segments. *JOURNAL OF AMBIENT INTELLIGENCE AND HUMANIZED COMPUTING* **2021**, *12*, 10145-10162, doi:10.1007/s12652-020-02779-1.
155. Piccini, J.P.; Stanelle, E.; Hylek, E.M.; Johnson, L.C.; Kanwar, R.; Lakkireddy, D.R.; Mittal, S.; Peacock, J.; Russo, A.M.; Soderlund, D.; et al. B-PO02-079 PERFORMANCE OF ATRIAL FIBRILLATION BURDEN PATTERNS DETECTED VIA INSERTABLE CARDIAC MONITOR FOR STROKE RISK STRATIFICATION. *Heart Rhythm* **2021**, *18*, S128, doi:https://dx.doi.org/10.1016/j.hrthm.2021.06.334.
156. Piorkowski, C.; Manyam, H.; Lakkireddy, D.; Byazrova, E.; Pavia, S.; Afzal, M.; John, J.; Qu, F.; Dawoud, F.; Davis, K.; et al. Effectiveness and performance of confirm RxTM SharpSenseTM technology: A multi-center retrospective analysis. *Journal of Arrhythmia* **2019**, *35*, 429-430, doi:https://dx.doi.org/10.1002/joa3.12273.
157. Pokaprakarn, T.; Kitzmiller, R.R.; Moorman, J.R.; Lake, D.E.; Krishnamurthy, A.K.; Kosorok, M.R. Sequence to Sequence ECG Cardiac Rhythm Classification Using Convolutional Recurrent Neural Networks. *IEEE JOURNAL OF BIOMEDICAL AND HEALTH INFORMATICS* **2022**, *26*, 572-580, doi:10.1109/JBHI.2021.3098662.
158. Pollock, K.G.; Sekelj, S.; Johnston, E.; Sandler, B.; Hill, N.R.; Ng, F.S.; Khan, S.; Nassar, A.; Farooqui, U. Application of a machine learning algorithm for detection of atrial fibrillation in secondary care. *International journal of cardiology. Heart & vasculature* **2020**, *31*, 100674, doi:https://dx.doi.org/10.1016/j.ijcha.2020.100674.
159. Qayyum, A.; Meriaudeau, F.; Chan, G.C.Y.; Ieee. Classification of Atrial Fibrillation with Pre-Trained Convolutional Neural Network Models. In Proceedings of the 2018 IEEE-EMBS CONFERENCE ON BIOMEDICAL ENGINEERING AND SCIENCES (IECBES), 2018; pp. 594-599.
160. Quartieri, F.; Cauti, F.M.; Calo, L.; Vicentini, A.; Huemer, M.; Ebrahim, I.; Kim, G.; Hutson, C.S.; Qu, F.; Dawoud, F.; et al. Retrospective analysis of confirm RxTM SharpSenseTM technology using real-world data from the SMART registry. *Journal of Arrhythmia* **2019**, *35*, 20, doi:https://dx.doi.org/10.1002/joa3.12266.
161. Rahul, J.; Sharma, L.D. Artificial intelligence-based approach for atrial fibrillation detection using normalised and short-duration time-frequency ECG. *BIOMEDICAL SIGNAL PROCESSING AND CONTROL* **2022**, *71*, doi:10.1016/j.bspc.2021.103270.
162. Rosa, G.; Laudato, G.; Colavita, A.R.; Scalabrino, S.; Oliveto, R. Automatic Real-time Beat-to-beat Detection of Arrhythmia Conditions. In Proceedings of the HEALTHINF: PROCEEDINGS OF THE 14TH INTERNATIONAL JOINT CONFERENCE ON BIOMEDICAL ENGINEERING SYSTEMS AND TECHNOLOGIES - VOL. 5: HEALTHINF, 2021; pp. 212-222.
163. Sahu, I.; Ukil, A.; Khandelwal, S.; Pal, A. LTH-ECG: Lottery Ticket Hypothesis-based Deep Learning Model Compression for Atrial Fibrillation Detection from Single Lead ECG On Wearable and Implantable Devices. *Annual International Conference of the IEEE Engineering in Medicine and Biology Society. IEEE Engineering in Medicine and Biology Society. Annual International Conference* **2022**, *2022*, 1655-1658, doi:https://dx.doi.org/10.1109/EMBC48229.2022.9871259.

164. Sandberg, E.L.; Grenne, B.L.; Berge, T.; Grimsmo, J.; Atar, D.; Halvorsen, S.; Fensli, R.; Jortveit, J. Diagnostic Accuracy and Usability of the ECG247 Smart Heart Sensor Compared to Conventional Holter Technology. *Journal of Healthcare Engineering* **2021**, *2021*, 5230947, doi:https://dx.doi.org/10.1155/2021/5230947.
165. Sasaki, K.; Mieda, R.; Hamada, S.; Hirao, K.; Sasano, T. Detection of atrial fibrillation using a deep learning with wearable pulse wave sensor. *Journal of Arrhythmia* **2019**, *35*, 163, doi:https://dx.doi.org/10.1002/joa3.12267.
166. Schack, T.; Safi Harb, Y.; Muma, M.; Zoubir, A.M. Computationally efficient algorithm for photoplethysmography-based atrial fibrillation detection using smartphones. *Annual International Conference of the IEEE Engineering in Medicine and Biology Society. IEEE Engineering in Medicine and Biology Society. Annual International Conference* **2017**, *2017*, 104-108, doi:https://dx.doi.org/10.1109/EMBC.2017.8036773.
167. Sideshwar, J.B.; Krishan, T.S.; Nagarajan, V.; Shanthakumar, S.; Vijayaraghavan, V. End-to-End Optimized Arrhythmia Detection Pipeline using Machine Learning for Ultra-Edge Devices. In Proceedings of the 20TH IEEE INTERNATIONAL CONFERENCE ON MACHINE LEARNING AND APPLICATIONS (ICMLA 2021), 2021; pp. 1501-1506.
168. Sims, H.; Clatworthy, P. Atrial fibrillation in stroke: Is using an ascod aetiological classification feasible in clinical practice? *European Stroke Journal* **2021**, *6*, 415, doi:https://dx.doi.org/10.1177/23969873211034932.
169. Sun, Y.; Yang, Y.Y.; Wu, B.J.; Huang, P.W.; Cheng, S.E.; Wu, B.F.; Chen, C.C. Contactless facial video recording with deep learning models for the detection of atrial fibrillation. *SCIENTIFIC REPORTS* **2022**, *12*, doi:10.1038/s41598-021-03453-y.
170. Tadi, M.J.; Mehrang, S.; Kaisti, M.; Lahdenoja, O.; Hurnanen, T.; Jaakkola, J.; Jaakkola, S.; Vasankari, T.; Kiviniemi, T.; Airaksinen, J.; et al. Comprehensive Analysis of Cardiogenic Vibrations for Automated Detection of Atrial Fibrillation Using Smartphone Mechanocardiograms. *IEEE SENSORS JOURNAL* **2019**, *19*, 2230-2242, doi:10.1109/JSEN.2018.2882874.
171. Taniguchi, H.; Takata, T.; Takechi, M.; Furukawa, A.; Iwasawa, J.; Kawamura, A.; Taniguchi, T.; Tamura, Y. Explainable Artificial Intelligence Model for Diagnosis of Atrial Fibrillation Using Holter Electrocardiogram Waveforms. *INTERNATIONAL HEART JOURNAL* **2021**, *62*, 534-539, doi:10.1536/ihj.21-094.
172. Tison, G.H.; Sanchez, J.M.; Ballinger, B.; Singh, A.; Olgin, J.E.; Pletcher, M.J.; Vittinghoff, E.; Lee, E.S.; Fan, S.M.; Gladstone, R.A.; et al. Passive Detection of Atrial Fibrillation Using a Commercially Available Smartwatch. *JAMA CARDIOLOGY* **2018**, *3*, 409-416, doi:10.1001/jamacardio.2018.0136.
173. Ukil, A.; Marin, L.; Mukhopadhyay, S.C.; Jara, A.J. AFSense-ECG: Atrial Fibrillation Condition Sensing From Single Lead Electrocardiogram (ECG) Signals. *IEEE SENSORS JOURNAL* **2022**, *22*, 12269-12277, doi:10.1109/JSEN.2022.3162691.
174. Wong, C.K.; Hai, J.J.; Lau, Y.-M.; Zhou, M.; Lui, H.-W.; Lau, K.K.; Chan, K.-H.; Mok, T.M.; Liu, Y.; Feng, Y.; et al. Protocol for Home-Based Solution for Remote Atrial Fibrillation Screening to Prevent Recurrence Stroke (HUA-TUO AF Trial): a randomised controlled trial. *BMJ open* **2022**, *12*, e053466, doi:https://dx.doi.org/10.1136/bmjopen-2021-053466.
175. Xia, Y.; Wulan, N.; Wang, K.; Zhang, H. Detecting atrial fibrillation by deep convolutional neural networks. *Computers in Biology and Medicine* **2018**, *93*, 84-92, doi:https://dx.doi.org/10.1016/j.compbiomed.2017.12.007.

176. Yao, Z.J.; Zhu, Z.Y.; Chen, Y.X.; Ieee. Atrial Fibrillation Detection by Multi-scale Convolutional Neural Networks. In Proceedings of the 2017 20TH INTERNATIONAL CONFERENCE ON INFORMATION FUSION (FUSION), 2017; pp. 1159-1164.
177. Yokokawa, M.; Ip, R.; Sheikh, A.; Castellani, M.; Ip, J. ACCURACY OF IMPLANTABLE CARDIAC MONITOR IN DIAGNOSING CARDIAC ARRHYTHMIAS: A PROSPECTIVE RANDOMIZED CLINICAL TRIAL COMPARING REVEAL LINQ TRURHYTHMTM VERSUS CONFIRM RX SHARPSENSETM. *Journal of the American College of Cardiology* **2020**, *75*, 440, doi:https://dx.doi.org/10.1016/S0735-1097%2820%2931067-6.
178. Yu, Y.; Wang, D. Detected Method of Paroxysmal Atrial Fibrillation Based on Mixed Expert Model. *Basic and Clinical Pharmacology and Toxicology* **2020**, *127*, 28, doi:https://dx.doi.org/10.1111/bcpt.13461.
179. Yue, Z.; Zhu, J.J.; Iop. Atrial Fibrillation Detection Based on EEMD and XGBoost. In Proceedings of the 2019 3RD INTERNATIONAL CONFERENCE ON MACHINE VISION AND INFORMATION TECHNOLOGY (CMVIT 2019), 2019.
180. Zalabarria, U.; Irigoyen, E.; Lowe, A. Diagnosis of atrial fibrillation based on arterial pulse wave foot point detection using artificial neural networks. *COMPUTER METHODS AND PROGRAMS IN BIOMEDICINE* **2020**, *197*, doi:10.1016/j.cmpb.2020.105681.
181. Zhu, L.; Nathan, V.; Kuang, J.; Kim, J.; Avram, R.; Olgin, J.; Gao, J. Atrial Fibrillation Detection and Atrial Fibrillation Burden Estimation via Wearables. *IEEE Journal of Biomedical and Health Informatics* **2022**, *26*, 2063-2074, doi:https://dx.doi.org/10.1109/JBHI.2021.3131984.
182. Baturova, M.A.; Sheldon, S.H.; Carlson, J.; Brady, P.A.; Lin, G.; Rabinstein, A.A.; Friedman, P.A.; Platonov, P.G. Electrocardiographic and Echocardiographic predictors of paroxysmal atrial fibrillation detected after ischemic stroke. *BMC Cardiovascular Disorders* **2016**, *16*, 209, doi:10.1186/s12872-016-0384-2.
183. Kallmünzer, B.; Bobinger, T.; Kopp, M.; Kurka, N.; Arnold, M.; Hilz, M.-J.; Schwab, S.; Köhrmann, M. Impact of Heart Rate Dynamics on Mortality in the Early Phase after Ischemic Stroke: A Prospective Observational Trial. *Journal of Stroke and Cerebrovascular Diseases* **2015**, *24*, 946-951, doi:https://doi.org/10.1016/j.jstrokecerebrovasdis.2014.12.009.
184. Skrebelyte-Strøm, L.; Rønning, O.M.; Dahl, F.A.; Steine, K.; Kjekshus, H. Prediction of occult atrial fibrillation in patients after cryptogenic stroke and transient ischaemic attack: PROACTIA. *EP Europace* **2022**, euac092, doi:10.1093/europace/euac092.
185. Haeusler, K.G.; Kirchhof, P.; Heuschmann, P.U.; Laufs, U.; Busse, O.; Kunze, C.; Thomalla, G.; Nabavi, D.G.; Röther, J.; Veltkamp, R.; et al. Impact of standardized MONitoring for Detection of Atrial Fibrillation in Ischemic Stroke (MonDAFIS): Rationale and design of a prospective randomized multicenter study. *American Heart Journal* **2016**, *172*, 19-25, doi:https://doi.org/10.1016/j.ahj.2015.10.010.
186. Haeusler, K.G.; Kirchhof, P.; Kunze, C.; Tütüncü, S.; Fiessler, C.; Malsch, C.; Olma, M.C.; Jawad-Ul-Qamar, M.; Krämer, M.; Wachter, R.; et al. Systematic monitoring for detection of atrial fibrillation in patients with acute ischaemic stroke (MonDAFIS): a randomised, open-label, multicentre study. *The Lancet Neurology* **2021**, *20*, 426-436, doi:https://doi.org/10.1016/S1474-4422(21)00067-3.

187. Poulsen, M.B.; Binici, Z.; Dominguez, H.; Soja, A.M.B.; Kruuse, C.; Hornnes, A.H.; Rasmussen, R.S.; Overgaard, K. Performance of short ECG recordings twice daily to detect paroxysmal atrial fibrillation in stroke and transient ischemic attack patients. *International Journal of Stroke* **2016**, *12*, 192-196, doi:10.1177/1747493016669883.
188. Huang, W.-Y.; Lee, M.; Sung, S.-F.; Tang, S.-C.; Chang, K.-H.; Huang, Y.-S.; Lee, J.-D.; Lee, T.-H.; Jeng, J.-S.; Chung, C.-M.; et al. Atrial fibrillation trial to evaluate real-world procedures for their utility in helping to lower stroke events: A randomized clinical trial. *International Journal of Stroke* **2020**, *16*, 300-310, doi:10.1177/1747493020938297.
189. Glotzer, T.V.; Daoud, E.G.; Wyse, D.G.; Singer, D.E.; Ezekowitz, M.D.; Hilker, C.; Miller, C.; Qi, D.; Ziegler, P.D. The Relationship Between Daily Atrial Tachyarrhythmia Burden From Implantable Device Diagnostics and Stroke Risk. *Circulation: Arrhythmia and Electrophysiology* **2009**, *2*, 474-480, doi:10.1161/CIRCEP.109.849638.
190. Wachter, R.; Gröschel, K.; Gelbrich, G.; Hamann, G.F.; Kermer, P.; Liman, J.; Seegers, J.; Wasser, K.; Schulte, A.; Jürries, F.; et al. Holter-electrocardiogram-monitoring in patients with acute ischaemic stroke (Find-AFRANDOMISED): an open-label randomised controlled trial. *The Lancet Neurology* **2017**, *16*, 282-290, doi:https://doi.org/10.1016/S1474-4422(17)30002-9.
191. Buck, B.H.; Hill, M.D.; Quinn, F.R.; Butcher, K.S.; Menon, B.K.; Gulamhusein, S.; Siddiqui, M.; Coutts, S.B.; Jeerakathil, T.; Smith, E.E.; et al. Effect of Implantable vs. Prolonged External Electrocardiographic Monitoring on Atrial Fibrillation Detection in Patients With Ischemic Stroke: The PER DIEM Randomized Clinical Trial. *JAMA* **2021**, *325*, 2160-2168, doi:10.1001/jama.2021.6128.
192. Brasier, N.; Engelter, S.; Kolbitsch, T.; Tabord, A.; Knobeloch, J.; Kühne, M.; Conen, D.; Traenka, C.; Kreutzberger, T.; Völlmin, G.; et al. The quest for indicators of paroxysmal atrial fibrillation in sinus rhythm – the DETECT AF trial. *Acta Cardiologica* **2019**, *74*, 301-307, doi:10.1080/00015385.2018.1493248.
193. Pereira, T.; Gadhoumi, K.; Ma, M.; Liu, X.; Xiao, R.; Colorado, R.A.; Keenan, K.J.; Meisel, K.; Hu, X. A Supervised Approach to Robust Photoplethysmography Quality Assessment. *IEEE Journal of Biomedical and Health Informatics* **2020**, *24*, 649-657, doi:https://dx.doi.org/10.1109/JBHI.2019.2909065.
194. Sung, S.-F.; Sung, K.-L.; Pan, R.-C.; Lee, P.-J.; Hu, Y.-H. Automated risk assessment of newly detected atrial fibrillation poststroke from electronic health record data using machine learning and natural language processing. *Frontiers in cardiovascular medicine* **2022**, *9*, 941237, doi:https://dx.doi.org/10.3389/fcvm.2022.941237.
